# Beyond Electrostatics: Anion‐π^+^ Orbital Hybridization Underpins High‐Performance Chloride Storage in Poly(arylamine) Organic Cathodes

**DOI:** 10.1002/advs.75896

**Published:** 2026-06-03

**Authors:** Tiantian She, Jiena Weng, Jie Wang, Qiao Xi, Yangyang Zhang, Zijie Zhang, Zongqiong Lin, Wei Huang

**Affiliations:** ^1^ State Key Laboratory of Flexible Electronics (LOFE) & Institute of Flexible Electronics (IFE) Shaanxi Key Laboratory of Flexible Electronics MIIT Key Laboratory of Flexible Electronics (KLoFE) Northwestern Polytechnical University Xi'an China; ^2^ State Key Laboratory of Organic Electronics and Information Displays & Institute of Advanced Materials (IAM) Nanjing University of Posts & Telecommunications Nanjing China; ^3^ Key Laboratory of Flexible Electronics & Institute of Advanced Materials Nanjing Tech University Nanjing China

**Keywords:** Anion‐π^+^ interactions, Cl^−^ storage, Covalent‐ionic hybrid, Organic electrode

## Abstract

The interactions between ionic charge carriers and host framework critically govern electrochemical reactions and ion‐storing performance, serving as a pivotal design consideration for energy storage devices. However, the fundamental understanding of the covalent‐ionic interactions between anion and oxidized π^+^‐framework remains limited thus far. Here we reveal the covalent‐ionic nature of anion‐π^+^ interactions between poly(arylamine)s (PAAs) and Cl^−^ anions. Cl^−^‐π^+^ complexes bearing rigid polymeric aryl‐substituted dihydrophenazine (PDPZ) exhibit both electrostatic interaction and distinct Cl^−^→π^+^ charge‐transfer orbital contribution, confirming the underlying hybrid covalent‐ionic nature of Cl^−^‐π^+^ interaction. The synergistic effect of Cl^−^‐π^+^ interaction and high electron delocalization capability of PDPZ^x+^ framework enables reversible Cl^−^ intercalation/deintercalation during multi‐electron redox, achieving a high anion storage capacity of 236 mAh g^−1^ and remarkable energy densities of 175 Wh kg^−1^ (Zn||PDPZ cell, in 30 m ZnCl_2_), alongside long calendar life as cathodes for Cl^−^‐based dual‐ion batteries (Cl‐DIBs). Spectroscopic evidence reveals dynamic evolution of vibrational modes and electronic structures from PDPZ to PDPZ^x+^·xCl^−^ complexes, demonstrating the entire π^+^‐framework participation and anion‐to‐π^+^ charge transfer during chloride storage. Our mechanistic insights into anion‐π^+^ interactions in Cl‐DIBs provide theoretical guidance for designing advanced anion‐storage organic cathodes and advance anion coordination chemistry.

## Graphical abstract

The fundamental nature of anion storage in p‐type organic cathodes is elucidated using rigid poly(arylamine) frameworks. This study reveals a hybrid covalent‐ionic mechanism for anion‐π^+^ interactions, driven by synergistic orbital hybridization and electrostatic attraction. This supramolecular paradigm enables the PDPZ cathode to achieve reversible multi‐electron chloride intercalation with high capacity and stability, guiding advanced organic mixed ionic‐electronic conductor design.

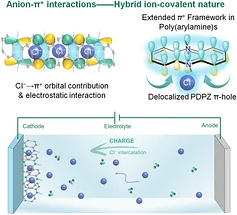

## 1 Introduction

Positive‐charge‐enriched atoms/groups of the electrode framework serve as binding sites for anionic charge carriers in electrochemical energy storage, forming covalent‐ionic bonds characterized by donor–acceptor covalency [1, 2]. However, anionic charge carriers typically exhibit larger ionic radii and more complex spatial configurations than their cationic counterparts, imposing more stringent demands on host electrode materials in terms of topological and electronic compatibility [3]. p‐Type redox‐active aromatic hydrocarbons (p‐RAHs), featuring with exceptional charge‐delocalization capability within their oxidized π^+^‐conjugated skeletons and tunable redox potential, represent highly promising anion‐hosting materials [4, 5]. Despite significant improvements in the electrochemical stability of p‐RAHs achieved through molecular engineering, optimizing their anion storage performance remains a critical challenge. This demands deeper probing of intrinsic storage mechanisms, specifically the covalent‐ionic interaction between p‐RAHs and anions.

The interactions between ionic charge carriers and the host frameworks directly govern their binding energy, ion‐transport barriers, and redox kinetics, making them pivotal in energy storage device design [6, 7, 8]. Current research, however, primarily focuses on multidimensional matching between the physicochemical properties of anions (e.g., Stokes radius, charge density, electrostatic surface potential) and the microstructural features of p‐RAHs frameworks (e.g., electron affinity, electron delocalization capability, molecular packing motifs) [9, 10, 11, 12]. For instance, anion charge density significantly dictates ion‐pair formation propensity, modulating charge‐transfer activation energy at p‐RAHs electrode interfaces [10]. To date, a thorough understanding of the nature of covalent‐ionic interaction between anions and p‐RAHs, particularly their oxidized π^+^‐conjugated backbones, remains limited. While isolated studies have demonstrated charge‐transfer between anions and electrophilic N^+^ sites in p‐RAHs [13], the prevailing model attributes anion adsorption exclusively to electrostatic attraction [9, 10]. Such assumption adopting electron localization model at discrete N^+^ sites and attributing the covalent‐ionic interactions solely to electrostatic forces oversimplifies the underlying chemistry. Due to the significant electron delocalization throughout the π^+^ framework, the bonding force of covalent‐ionic bonds should be governed by the interactions between the anions and the entire electron‐deficient π^+^‐framework rather than only one electrophilic atom/group [2, 14]. From a supramolecular chemistry perspective, the inherent electron‐deficient nature of the π^+^‐framework induces intricate anion‐π^+^ interactions [15]. Such interactions encompass coupled electrostatic attraction, frontier orbital overlap, induced polarization and so on, resulting in charge transfer between anions and the π^+^ systems [16]. Therefore, to break away from the oversimplified electrostatic interaction model, deeper investigations are required to elucidate the nature of interactions between anions and the π^+^‐framework of p‐RAHs as well as their role for anion storage, which would guide the rational design of next‐generation high‐performance organic cathode materials and advance anion coordination chemistry.

Herein, we investigate the covalent‐ionic nature of anion‐π^+^ interactions by electrochemically p‐doping linear p‐RAHs with strongly nucleophilic chloride ions. The Cl^−^‐π^+^ interactions between Cl^−^ anions and the positively charged aryl‐substituted dihydrophenazine (DPZ) moieties of polymeric PDPZ are simultaneously governed by both of electrostatic interactions and π^+^‐orbital interactions. Benefiting from the efficient π‐hole delocalization of conjugation extended π^+^ framework as well as Cl^−^‐π^+^ interactions with hybrid ion‐covalent nature, PDPZ electrode delivers a high Cl^−^ storage capacity of 236 mAh g^−1^ and remarkable energy densities of 175 Wh kg^−1^ as the cathode of Cl^−^‐based dual‐ion batteries (Cl‐DIBs). In situ and ex situ spectroelectrochemical characterizations identify the charge carrier species and elucidate the critical bonding mechanisms underlying redox‐driven anion intercalation/deintercalation, with particular emphasis on the dynamic evolution of Cl^−^‐π^+^ interactions during electrochemical cycling.

## 2 Results and Discussion

### 2.1 Molecular and Electronic structures

**FIGURE 1:**
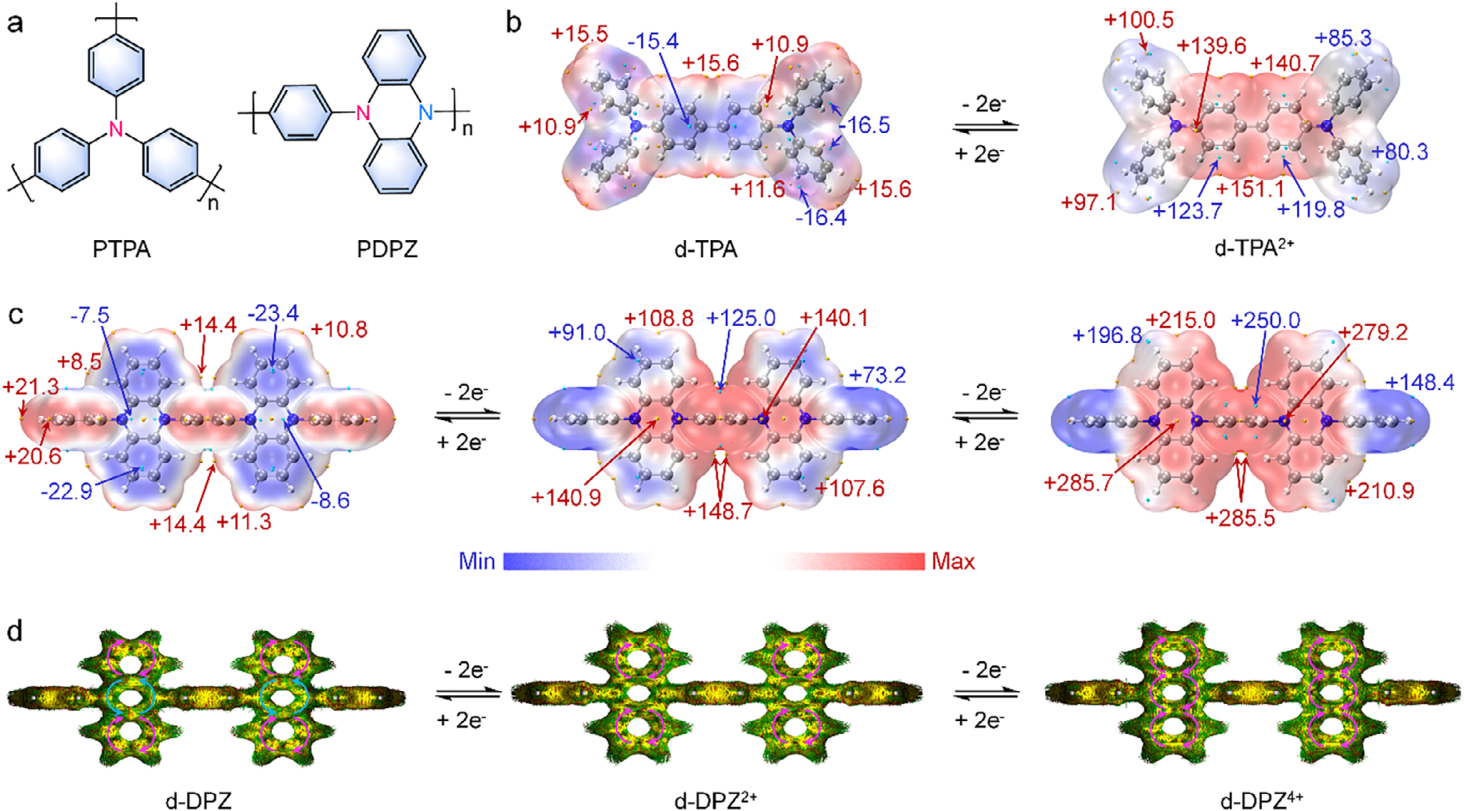
Electronic structures of PTPA and PDPZ elucidated by their dimer models. (a) Chemical structures of PTPA and PDPZ. Molecular electrostatic potential distribution of (b) d‐TPA and (c) d‐DPZ at different oxidation states. (d) ACID plots of d‐DPZ, d‐DPZ^2+^, and d‐DPZ^4+^. The orange‐red and deep sky‐blue arrows represent the clockwise (aromatic) and anticlockwise (antiaromatic) ring currents, respectively.

(Poly(arylamine)s (PAAs), as prototypical p‐RAHs with quaternary nitrogen redox centers [17], have emerged as cathodes with exceptional anion‐storage capacity for nonaqueous dual‐ion batteries. Their conjugated backbone enables electrons and/or polarons delocalization in oxidized PAAs^x+^, creating delocalized π‐holes that facilitate reversible anion binding without structural reorganization during redox processes. However, PAAs^x+^ radicals exhibit limited stability under aqueous conditions due to water/oxygen susceptibility [18]. Building on the understanding that electron‐deficient aromatic systems enhance anion‐π interactions (particularly in cationic rings where π^+^ acidity maximizes binding affinity), we hypothesized that tailoring anion‐π^+^ interactions could extend the applicability of PAA^x+^ cathodes to aqueous electrolytes. To test this hypothesis and probe the physical origin of anion‐π^+^ interactions, we engineered two PAAs with distinct π‐electron delocalization capabilities and employed chloride ion (Cl^−^)—a fundamental monoatomic charge carrier—as the model anion. Comparative studies utilized PDPZ, a DPZ‐incorporated PAA with rigid π‐delocalized architecture, against conventional propeller‐shaped poly(triphenylamine) (PTPA) (Figure 1a). The conformationally locked DPZ moiety enables the effective participation of nitrogen lone‐pair electrons (perpendicular to the planar configuration of phenazine) in p‐orbital delocalization, achieving extended π‐conjugation. In contrast, steric hindrance in TPA units enforces a non‐planar geometry, diminishing π‐orbital participation of nitrogen lone pair electrons (Figure S1a). Significant diverse electronic structures between TPA and DPZ moieties create an ideal platform for tailoring anion‐PAA^x+^ interactions through electronic structure modulation. The synthesis and chemical structure characterization of PTPA and PDPZ are provided in Schemes S1 and S2 and Figures S2–S5.

Density functional theory (DFT) calculations were conducted on dimers (denoted as d‐TPA and d‐DPZ) to probe the intrinsic electronic structures of the corresponding polymers (Figure S1b). Frontier molecular orbital analysis of the neutral dimers reveals enhanced conjugation in d‐DPZ, where the highest occupied molecular orbital (HOMO) exhibits delocalized electron density across both phenazine planes, contrasting with the inhomogeneous distribution on individual benzene rings in d‐TPA (Figure S6). The elevated HOMO level of d‐DPZ (−4.72 eV) indicates reduced ionization potential to facilitate hole extraction during the initial two‐electron (one electron for a phenazine ring) oxidation process. The conformational locking in d‐DPZ promotes effective conjugation through N lone pair electron contribution to π‐electron orbital overlap, yielding a narrowed energy gap (*E*_g_ = 3.55 eV). To identify anion‐binding interactions in d‐TPA and d‐DPZ, molecular electrostatic potential (ESP) maps are introduced to visualize the charge distribution of d‐TPA and d‐DPZ across oxidation states (neutral state: d‐TPA/d‐DPZ, +2 valence state: d‐TPA^2+^/d‐DPZ^2+^; +4 valence state: d‐DPZ^4+^) (Figure 1b,c). Neutral species show divergent electron‐rich domains, d‐DPZ exhibiting an electron‐rich pyrazine core vs. biphenyl‐centered electron enrichment in d‐TPA (blue regions, Figure 1b). Quantitatively, neutral d‐DPZ exhibits more negative ESP minimum (−23.4 kcal mol^−1^), indicating significantly enhanced electron‐donating capacity relative to neutral d‐TPA. Oxidation induces polarization inversion for both PAAs, converting original electron‐rich zones into pronounce electron‐poor regions with ESP maxima. While d‐TPA^2+^ retains electron‐deficient region confinement to biphenyl units, d‐DPZ^2+^/d‐DPZ^4+^ exhibits phenazine‐wide delocalization extending to adjacent benzene rings. Two ESP extremum points (+140.9 and +285.7 kcal mol^−1^ for d‐DPZ^2+^ and d‐DPZ^4+^, respectively; red arrows) emerge atop pyrazine rings in both oxidized d‐DPZ derivatives, suggesting enhanced electrophilicity at these regions that enables electrostatically favored anion‐binding selectivity.

Aromatic stabilization through electron delocalization in the neutral and oxidized dimers was quantified by nuclear independent chemical shift (NICS(1)zz) [19, 20], and visualized via anisotropy of induced current density (ACID) [21, 22], uncovering distinct oxidation‐state‐dependent aromatic evolution. Neutral d‐TPA demonstrates characteristic aromaticity with negative NICS(1)zz values of −24–−21 ppm and clockwise ACID current in benzene rings (Figures S7 and S8). Upon oxidation, d‐TPA^2+^ exhibits diminished aromatic characteristics (NICS(1)zz = −8.43 ppm) in the central biphenyl fragment, reflecting oxidation‐induced π‐system disruption. Owing to the involvement of nitrogen lone pair electrons into π‐conjugation, neutral d‐DPZ manifests pyrazine‐centered antiaromaticity (NICS(1)zz = +28.65/+27.62 ppm) with counterclockwise ACID current (Figure 1d), along with attenuated aromaticity (NICS(1)zz = −12.99/−13.89 ppm) in adjacent benzene rings fused with pyrazine through π‐electron redistribution. Stepwise electronic reorganization upon oxidation results in sequential aromaticity transitions of pyrazine rings in oxidized d‐DPZ derivatives: antiaromatic (d‐DPZ) → nonaromatic (d‐DPZ^2+^) → aromatic (d‐DPZ^4+^), aligning with thermodynamic stabilization [23]. Global electron delocalization across the phenazine fragments stabilizes π^+^ cations of d‐DPZ^2+^ and d‐DPZ^4+^ through enhanced π‐orbital overlap. Progressive negative shifts in NICS(1)zz values for d‐DPZ^2+^ (−15.3 ppm) and d‐DPZ^4+^ (−18.7 ppm), driven by expanded electron delocalization, demonstrate stepwise enhancement of global aromaticity and conjugation upon oxidation. Such aromaticity evolution adheres to Hückel's rule, endowing d‐DPZ^4+^ intermediates with exceptional thermodynamic stability, which is consistent with previous reports [24, 25].

### 2.2 Interactions Between Cl^−^ and PAA^x+^ Frameworks

**FIGURE 2:**
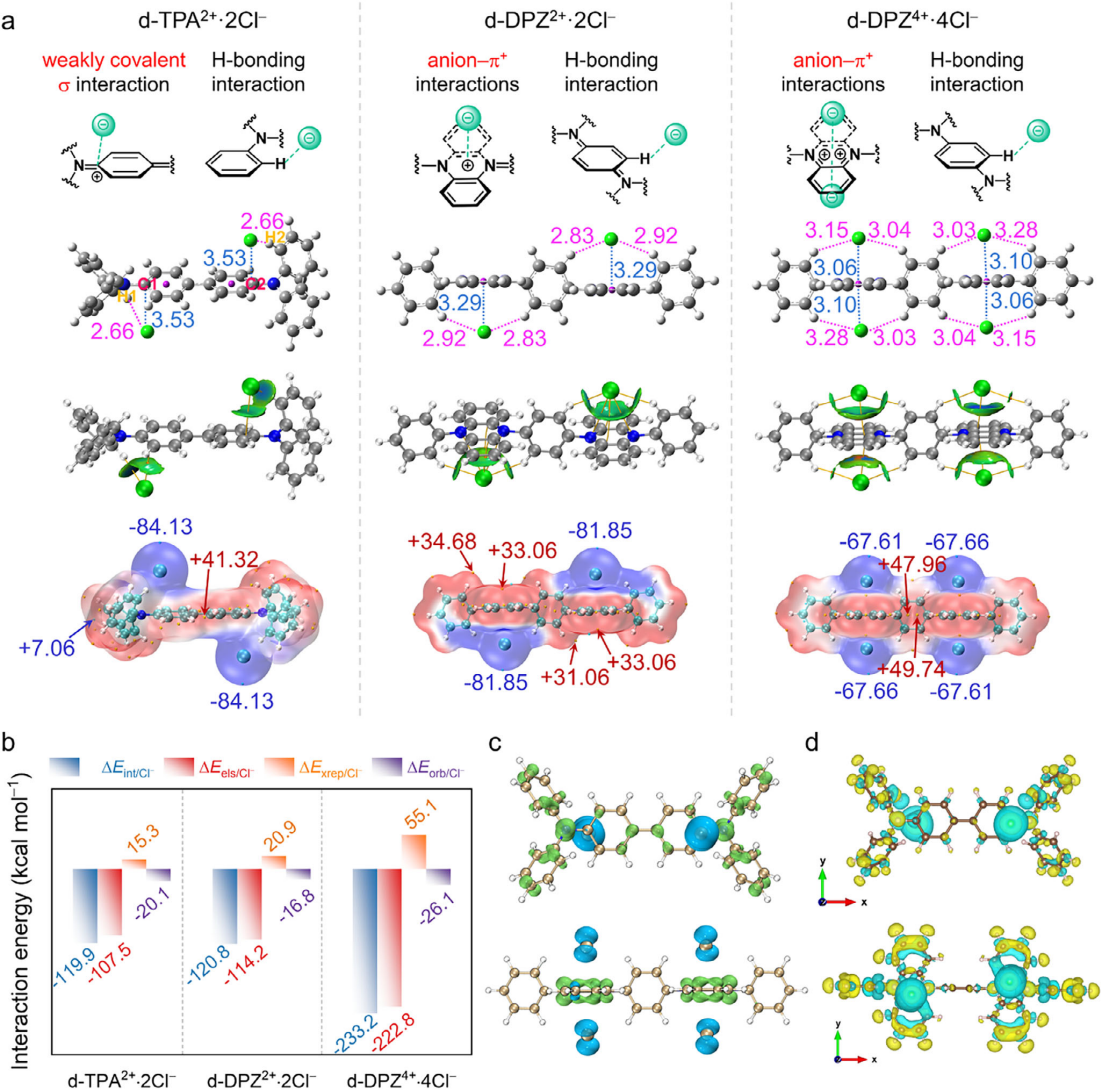
Interactions between anions and π^+^ framework in PAA^x+^·xCl^−^ complexes. (a) Optimized geometries (the second row), IGMH isosurface maps (the third row, sign(*λ*_2_)ρ colored isosurfaces of *δ*g^inter^ = 0.004 a.u.), and molecular ESP distribution (the bottom row) of PAA^x+^·xCl^−^ complexes. The top row schematically represents the involved interaction types in each anion‐π^+^ complexes. Representative Cl^−^···H/C/ring‐centroid distances (Å) are provided for each optimized anion‐π^+^ complexes. In the IGMH map, orange spheres and yellow lines correspond to BCPs and bond paths, respectively. Electron density values at these BCPs are provided in Table S2. (b) EDA‐NOCV analyses of d‐TPA^2+^·2Cl^−^, and d‐DPZ^x+^·xCl^−^ (x = 2, 4) yield interaction energy components (kcal mol^−1^). $\Delta E_{\mathrm{int}/Cl^{-}}$, $\Delta E_{\mathrm{els}/Cl^{-}}$, and $\Delta E_{\mathrm{orb}/Cl^{-}}$ represent the the average interaction energy per chloride of total interaction, electrostatic, exchange‐repulsion, and orbital interaction energies, respectively. (c) The isosurface of NOCV pair 1 density and (d) EDD of d‐TPA^2+^·2Cl^−^ (top) and d‐DPZ^4+^·4Cl^−^ (bottom).

We employed supramolecular analysis to elucidate the interactions between positively charged PAAs and anions. Here, three PAA^x+^·xCl^−^ ion pairs (denoted as d‐TPA^2+^·2Cl^−^, d‐DPZ^2+^·2Cl^−^, and d‐DPZ^4+^·4Cl^−^) are adopted for anion‐π^+^ interactions analysis according to their formation Gibbs free energy (Figure S9 and Table S1). The optimized geometries of PAA^x+^·xCl^−^ ion pairs (Figure 2a, the second row) demonstrate Cl^−^ anions occupying ESP maxima in their cationic frameworks. The triphenylamine propeller motif persists in d‐TPA^2+^·2Cl^−^, with nucleophilic Cl^−^ counterions located at aromatic peripheries (specifically positioned over the terminal C1/C2 atoms of biphenyl group with C···Cl bonding distance of 3.53 Å), indicating an off‐center Cl^−^ binding geometry. Such off‐center geometry suggests there exist weakly covalent σ interactions between the cationic d‐TPA^2+^ and Cl^−^ counterions, forming a weakly covalent donor‐π‐acceptor (donor to π‐acceptor) complex [26, 27]. Weak hydrogen bonding interactions exist between the Cl^−^ ions and two of the peripheral phenyl groups with H···Cl bond length of 2.66 Å. Independent gradient model based on Hirshfeld partition (IGMH)analysis [28] visually confirmed that the weak interaction is dominated by the weakly covalent σ interactions and H···Cl hydrogen bonding (Figure 2a, the third row). In contrast to d‐TPA^2+^·2Cl^−^, the d‐DPZ^2+^·2Cl^−^ complex adopts a centroid‐coordinated geometry with two Cl^−^ anions positioned 3.29 Å above distinct pyrazine ring centroids — a configuration consistent with the ESP analysis, mirroring classical anion‐π^+^ systems where anions vertically coordinate electron‐deficient aromatic cores. Remarkably, oxidation to d‐DPZ^4+^·4Cl^−^ induces face‐on dual Cl^−^ coordination per phenazine unit, forming a stabilized sandwich architecture (phenazine unit is sandwiched between two Cl^−^ anions). Cl^−^ anions maintain near‐centroid binding to pyrazine rings with shortened coordination distances (3.06 and 3.10 Å, the distances between the Cl^−^ and the pyrazine ring centroids), indicating enhancement of anion‐π^+^ interactions in the d‐DPZ^4+^·4Cl^−^ species. IGMH analysis of d‐DPZ^2+^·2Cl^−^ and d‐DPZ^4+^·4Cl^−^ reveals interfacial green isosurfaces between Cl^−^ anions and pyrazine rings, confirming anion‐π^+^ interactions.

Atoms in molecules (AIM) analysis [29, 30] showed that the sum of electron density (*ρ*(r)) and its Laplacian (*∇* ^2^ *ρ*(r)) at the bond critical points (BCPs) between Cl^−^ ions and PAA^x+^ gradually increases: d‐TPA^2+^·2Cl^−^ < d‐DPZ^2+^·2Cl^−^ < d‐DPZ^4+^·4Cl^−^ (Table S2). The electron‐deficient of pyrazine critically stabilize Cl^−^ anions in d‐DPZ^x+^·xCl^−^. With increasing oxidation state, the sum of *ρ*(r) and *∇* ^2^ *ρ*(r) value progressively rise from 0.077 to 0.090 a.u. and 0.232 to 0.264 a.u., respectively, indicating strengthened anion‐π^+^ coupling, which is also consistence with interaction region indicator (IRI) analyses (Figures S10 and S11). Furthermore, H···Cl hydrogen bonding weakens in d‐DPZ^4+^·4Cl^−^ vs. d‐DPZ^2+^·2Cl^−^, evidenced by elongated H···Cl distances and decreased *ρ*(r_H‐Cl_) values.

To elucidate Cl^−^‐coordination induced electronic reorganization and aromatic stabilization, we compared frontier molecular orbitals, ESP distributions, and NICS(1)zz indices between PAA^x+^·xCl^−^ complexes and PAA^x+^ cations. DFT calculations demonstrate Cl^−^ coordination induces significant HOMO upshifts in PAA^x+^·xCl^−^ complexes vs. isolated cations (ΔHOMO = +0.15/+0.22/+1.15 eV for d‐TPA^2+^·2Cl^−^/d‐DPZ^2+^·2Cl^−^/d‐DPZ^4+^·4Cl^−^; Figures S12 and S13), attributed to strong electron donation from Cl^−^ anions to π^+^ skeleton. The largest HOMO elevation drives pronounced bandgap narrowing (Δ*E*_g_ = 0.44 eV) in d‐DPZ^4+^·4Cl^−^ compared to its uncoordinated d‐DPZ^4+^ counterpart. Both d‐TPA^2+^·2Cl^−^ and d‐DPZ^4+^·4Cl^−^ exhibit HOMO delocalization across π^+^‐skeleton to Cl^−^ anions, evidencing Cl^−^ participation in multielectron redox conjugation. Cl^−^‐mediated charge equilibration significantly attenuates the ESP positive extremum in PAA^x+^·xCl^−^ complexes vs. isolated cations (Figure 2a and Figure S14). For d‐DPZ^x+^·xCl^−^, this charge equilibration further leads to more homogeneous delocalization of positive ESP across the entire π‐cationic core—an observation that reflects stronger π‐backbone electron delocalization via anion coordination, relative to that in d‐TPA^2+^·2Cl^−^. The charge complementarity significantly stabilizes the Cl^−^‐coordinated systems through electrostatic reinforcement. NICS(1)zz analysis reveals minimal aromaticity variation in d‐DPZ^4+^·4Cl^−^ (*vs*. d‐DPZ^4+^), compared to dramatic shifts in d‐TPA^2+^·2Cl^−^ (vs. d‐TPA^2+^), demonstrating higher stability of the π‐cationic d‐DPZ^4+^ framework during Cl^−^ coordination (Figure S7). The preserved aromatic character and rigid π‐electron delocalization enable robust anion binding in d‐DPZ^4+^·4Cl^−^ through strong anion‐π^+^ interactions.

Energy decomposition analysis (EDA) [31]. elucidates the physical nature of the interactions between Cl^−^ anions and PAA^x+^ (Figure 2b and Table S3). To compare across complexes with different numbers of chloride ions and total charges, the interaction energies were normalized by the number of coordinated chloride ions [32]. The d‐DPZ^2+^·2Cl^−^ complex exhibits a slightly more favorable average total interaction energy $\left(\Delta E_{\mathrm{int}/Cl^{-}}\right)$ = −120.8 kcal mol^−1^) than d‐TPA^2+^·2Cl^−^ (−119.9 kcal mol^−1^). Notably, the $\Delta E_{\mathrm{int}/Cl^{-}}$ of d‐DPZ^4+^·4Cl^−^ (−233.23 kcal mol^−1^) is approximately double those of both d‐DPZ^2+^·2Cl^−^ and d‐TPA^2+^·2Cl^−^, indicating the strongest Cl^−^−PAA^x+^ interaction among the three studied species. This value also surpasses those of control systems involving polyatomic anions (e.g., TFSI^−^ and OTf^−^). Decomposition of the attractive interaction per chloride $\left(\Delta E_{\mathrm{att}/Cl^{-}}\right)$ shows that the average electrostatic $\left(\Delta E_{\mathrm{els}/Cl^{-}}\right)$ and orbital $\left(\Delta E_{\mathrm{orb}/Cl^{-}}\right)$ interaction energies for d‐DPZ^4+^·4Cl^−^ are −222.8 and −26.15 kcal mol^−1^, respectively, exceeding those of all other studied PAA^x+^·x(anion)^−^ systems. The orbital contribution $\left(\Delta E_{\mathrm{orb}/Cl^{-}}/\Delta E_{\mathrm{att}/Cl^{-}}\right)$ accounts for 11.8% and 10.0% for d‐DPZ^2+^·2Cl^−^ and d‐DPZ^4+^·4Cl^−^, respectively, while the electrostatic contribution $\left(\Delta E_{\mathrm{els}/Cl^{-}}/\Delta E_{\mathrm{att}/Cl^{-}}\right)$ rises to 80.6% and 85.4%. The contributions of covalent and ionic character were further quantified by the ratios Δ*E*_orb_/(Δ*E*_els_ + Δ*E*_orb_) and Δ*E*_els_/(Δ*E*_els_ + Δ*E*_orb_), respectively [33]. For the d‐DPZ^4+^·4Cl^−^ complex, this gives 10.5% covalent and 89.5% ionic character (Table S3), indicating that the interaction maximizes electrostatic stabilization while retaining measurable covalent character. Extended transition state‐natural orbitals for chemical valence (ETS‐NOCV) is further employed to dissect Δ*E*_orb_ contributions (Figure 2c and Figure S15). ETS‐NOCV mapping reveals electron density rearrangements with charge transfer (CT) flux from Cl^−^ lone‐pairs to antibonding orbitals of PAA^x+^ acceptors. In the case of d‐TPA^2+^·2Cl^−^, significant CT from Cl^−^ ions to d‐TPA^2+^ framework occurs, with electron density accumulation at the N and terminal phenyl carbon atoms, suggesting limited electron delocalization capability within the d‐TPA^2+^ framework upon Cl^−^ coordination. In d‐DPZ^4+^·4Cl^−^ complex, pronounced electron density accumulation across phenazine rings confirms orbital‐mediated Cl^−^→ π^+^ electron donation. EDA and ETS‐NOCV results demonstrate the Cl^−^‐π^+^ interactions in d‐DPZ^x+^·xCl^−^ complexes feature hybrid ion‐covalent character, distinct from classical noncovalent anion‐π interaction where charge‐transfer is negligible [16].

Inspired by that covalent‐ionic bonds in inorganic/graphite cathode exhibited donor‐acceptor covalency [34, 35], we conducted atomic dipole corrected Hirshfeld (ADCH) [36] and electron‐density difference (EDD) [37, 38] to elucidate charge transfer between Cl^−^ and the positively charged regions in PAA^x+^·xCl^−^ complexes. The negative ADCH change (−0.736–−1.592 e) of the PAA^x+^ fragment in the PAA^x+^Cl^−^ complexes relative to the isolated cation indicates electron accumulation in PAA^x+^ upon chloride coordination (Figure S16 and Table S4). The average transfer charge per Cl^−^ in d‐DPZ^2+^·2Cl^−^ and d‐DPZ^4+^·4Cl^−^ is 0.368 and 0.398 e, respectively, which is slightly lower than that in d‐TPA^2+^·2Cl^−^ (0.404 e). Despite these similar magnitudes, the spatial distribution of accumulated charge within PAA^x+^ fragments differs markedly across the systems, which is governed by the π‐delocalization capacity of the molecular backbone. In contrast to the high ADCH charge fluctuations (especially in terminal diphenylamine groups) of d‐TPA^2+^ moiety in d‐TPA^2+^·2Cl^−^, the d‐DPZ^x+^ fragments in complexes maintains uniform ADCH distribution due to the phenazine‐mediated π‐delocalization. The electron transfer behavior is further corroborated by EDD analysis, which visualizes charge depletion (colored cyan) around Cl^−^ anions and charge accumulation (colored yellow) at electrophilic PAA^x+^ fragment (Figure 2d and Figure S17). In d‐DPZ^x+^·xCl^−^ complexes, the charge enriched region delocalizes across the entire phenazine rings, indicating that the π^+^‐skeleton participates in Cl^−^‐π^+^ interaction (as evidenced by ETS‐NOCV). In contrast, d‐TPA^2+^·2Cl^−^ forms discrete electron‐transfer bridges between Cl^−^ and C1/C2 atoms in terminal benzene rings, consistent with weakly covalent *σ* interaction [39]. Localized electron accumulation on these diphenylamine terminal sites would result in unstable radical cations and unfavorable parasitic reactions.

The above theoretical analysis of PAA^x+^·xCl^−^ complexes provides an example where anion storage via ionic‐covalent interactions dominated by electrostatic attraction with measurable charge transfer. Specifically, interactions between strongly nucleophilic Cl^−^ anions and highly electron‐deficient PAA^x+^ (d‐TPA^2+^ and d‐DPZ^4+^) frameworks exhibit partial covalent character. In contrast, DFT investigations of polyatomic anions (OTf^−^ and TFSI^−^) interacting with PAA^x+^ frameworks show negligible charge transfer, indicating predominantly noncovalent binding (Figures S18 and S19). IGMH and AIM analysis (Figure S18) identify single‐point interactions in PAA^x+^·xCl^−^ vs. multicenter interactions in PAA^x+^·xOTf^−^/xTFSI^−^ systems [40]. Multicenter‐bonded associations reduce orbital interaction contributions while enhancing dispersion interactions between polyatomic anions and PAA^x+^ frameworks, as demonstrated by EDA (Table S3). Notably, ETS‐NOCV analysis demonstrates minimal electron accumulation on both d‐TPA^2+^ and d‐DPZ^4+^ frameworks in PAA^x+^·xOTf^−^/xTFSI^−^ systems, indicating that the coordination of multivalent anions through Coulomb attraction minimally perturbs the intrinsic charge density distribution of PAA^x+^ frameworks (Figure S19).

In light of the above findings, anion‐PAA^x+^ interactions are governed by both anion nucleophilicity and the electron delocalization capability of PAA^x+^ frameworks. Weakly nucleophilic polyatomic anions neutralize the positive charge of PAA^x+^ frameworks through noncovalent bonding. Their coordination exhibits negligible dependence on the frameworks' electron delocalization capability, predicting similar storage capacities for weakly nucleophilic anions across diverse PAA^x+^ systems. Conversely, for systems involving strongly nucleophilic halide ions (e.g., Cl^−^), the electron delocalization capability of the PAA^x+^ framework dictates performance due to charge‐transfer covalent bonding. Post‐charge‐transfer, the superior electron delocalization capability of d‐DPZ^x+^ frameworks enables stable and reversible Cl^−^ intercalation/deintercalation, while the limited delocalization capacity of d‐TPA^2+^ likely degrades performance. Our work thus advances beyond neutral‐state PAA delocalization analysis, uniquely accounting for both anion‐coordination‐induced charge transfer and the resultant framework electron delocalization capability.

### 2.3 Cl^−^ Storage Capability

**FIGURE 3:**
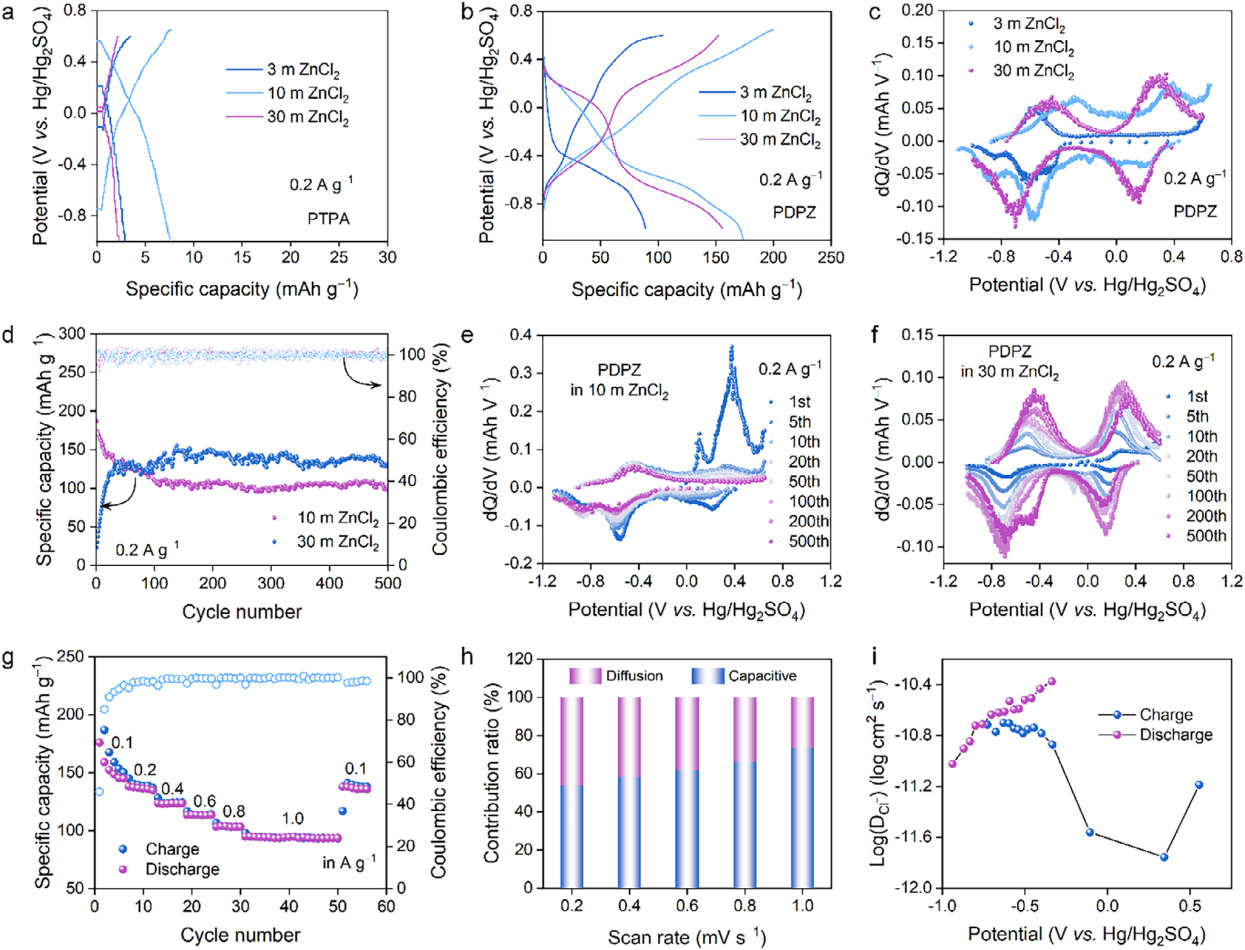
Anion storage capability of PTPA and PDPZ. Galvanostatic charge‐discharge (GCD) curves of (a) PTPA and (b) PDPZ in aqueous ZnCl_2_ (3, 10, and 30 m) electrolytes under a current density of 0.2 A g^‒1^. (c) d*Q*/d*V* profiles of PDPZ in different electrolytes at 0.2 A g^‒1^. (d) Cycling performance of PDPZ in 10 and 30 m ZnCl_2_ at 0.2 A g^‒1^. d*Q*/d*V* trajectory of PDPZ during cycling in (e) 10 m and (f) 30 m ZnCl_2_ at 0.2 A g^‒1^. (g) Rate performance of PDPZ at different current densities ranging from 0.1 to 1 A g^‒1^. (h) Diffusion/capacitive contributions in PDPZ quantified across scan rates. (i) Ion diffusion coefficient in PDPZ electrode determined from GITT.

To probe the role of interactions between Cl^−^ and PAA^x+^ frameworks on electrochemical Cl^−^ storage, we systematically evaluated PTPA and PDPZ electrodes in concentrated aqueous ZnCl_2_ electrolytes (up to 30 m “water‐in‐salt”, m: mol kg^−1^). The adoption of concentrated electrolyte aims to eliminate parasitic reactions (such as water decomposition) and enable unambiguous assessment of the anion‐π^+^ effect on electrochemical performance by minimizing the interference of free water [41]. PTPA electrodes exhibit negligible Cl^−^ storage capacity with discharge capacities < 15 mAh g^−1^ in all studied ZnCl_2_ electrolytes (Figure 3a), even in 30 m ZnCl_2_ after activation for 500 cycles (Figure S20). On the contrary, the PDPZ electrodes deliver superior Cl^−^ storage with maximum discharge capacities ranging from ca. 155 to 174 mAh g^−1^ (@0.2 A g^−1^, Figure 3b), achieving ∼86% utilization of theoretical specific capacity. This performance is facilitated by partially covalent Cl^−^‐π^+^ interactions with measurable charge‐transfer and the outstanding electron delocalization of PDPZ^x+^ skeleton post‐charge‐transfer. The marked contrast in Cl^−^ storage performance between PTPA and PDPZ electrodes underscores the critical role of electron delocalization capability within the positively charged polymer framework after the incorporation of partially covalent‐bonded anions. Galvanostatic charge‐discharge (GCD) and differential capacity (d*Q*/d*V*) profiles reveal two distinct redox stages with paired anodic/cathodic peaks, respectively, in both 10 and 30 m ZnCl_2_ electrolytes (Figure 3c). Each stage delivers ∼50% of total capacity, confirming theoretically predicted two‐step single‐electron redox reactions. The rigid, extended π‐conjugated backbone of PDPZ facilitates extensive π‐hole delocalization, enhancing anion‐π^+^ complexation to enable successful two‐electron oxidation. The high‐potential stage (0.2–0.4 V vs. Hg/Hg_2_SO_4_, second electron transfer) vanishes in 3 m ZnCl_2_, which might be attributed to the undesired side reactions between the highly active radical cation (PDPZ^x+^) and adsorbed H_2_O/OH^−^ from electrolyte [42, 43].

Further studies on anion dependence shows that PTPA and PDPZ electrodes deliver specific capacities over 80 and 120 mAh g^−1^ respectively for all studied chaotropic polyatomic anions (TFSI^−^, OTf^−^ and ClO_4_^−^; Figure S21). In these electrolytes, the superior specific capacities of PDPZ than those of PTPA originates from its higher active site density (n = 2 per DPZ unit vs. n = 1 for TPA, corresponding to theoretical specific capacities of 209 and 109 mAh g^−1^). The universality of PTPA and PDPZ in storing weakly nucleophilic polyatomic anions confirms that anion storage driven by non‐covalent electrostatic interactions is independent of the charge delocalization capability of the oxidized host frameworks. Additionally, the high polyatomic anion storage capacity of PTPA electrodes indicates that the previously proposed undesirable oxygen evolution at the active N^+^ sites in aqueous electrolytes is not the dominated cause of its ultra‐low Cl^−^ storage capacity. Instead, DFT analysis of the d‐TPA^2^^+^·2Cl^−^ complex, encompassing supramolecular interactions, geometric configuration, aromaticity, and charge transfer (via ADCH, EDD, and ETS‐NOCV methods), reveals that the poor Cl^−^ storage stems from insufficient charge delocalization within the conjugated framework. This deficiency will intensify charge fluctuation and electron density accumulation at the diphenylamine termini, leading to unstable radical cations and facilitating parasitic reactions. These results experimentally underscore that for ion storage governed by hybrid ionic–covalent interactions, strong charge transfer from the anion must be compensated by sufficient charge delocalization within the cationic aromatic framework, which is essential for stabilizing the resulting complex.

Long‐term GCD cycling analyses in the moderately concentrated 10 m ZnCl_2_ confirms the second‐electron redox degradation mechanism (Figure 3d,e and Figures S22 and S23). The PDPZ electrode undergoes an initial sharp capacity fade coupled with Coulombic efficiency (CE) rises from 63% to ∼98% during the first 10 cycles (Figure S22a,c), which is ascribed to the irreversible solid–electrolyte interphase (SEI) formation and dynamic evolution (see supplement note to Figures S22 and S23) [45, 46]. Subsequent cycles show gradual capacity decay with negligible CE loss, stabilizing at ∼100 mAh g^−1^ after 100 cycles (Figure 3d and Figure S22b,d). GCD profiles and corresponding d*Q*/d*V* evolution of this stage (Figure 3e and Figure S22b) reveal progressive capacity fading at the high‐potential plateau (0.2–0.4 V vs. Hg/Hg_2_SO_4_), which is mainly attributed to undesired side reactions between the highly reactive radical cation (PDPZ^x+^) and oxygenic intermediates (e.g., ·OH, ·O_2_^2−^) via free‐water oxidation at elevated potentials. The retained capacity (∼100 mAh g^−1^) originates predominantly from the low‐potential redox plateau. Such degradation can be inhibited using “water‐in‐salt” electrolyte (30 m ZnCl_2_) as the elimination of free‐water, where only ∼15% capacity fading is observed, and both redox stages are maintained even after 500 charge/discharge cycles at 0.2 A g^−1^ (Figure 3f and Figure S24), despite requiring ∼50‐cycle activation for full redox accessibility due to higher viscosity and reduced ionic conductivity [47, 48]. The corresponding calendar life reaches as long as 775 h, rivaling that of state‐of‐the‐art p‐type organic cathodes for polyatomic anions (Figure S29 and Table S5) [49, 50], underscoring the potential of PDPZ as a highly stable Cl^−^‐storage cathode, irrespective of the stability of the Zn anode.

We subsequently evaluated anion intercalation kinetics in PDPZ electrodes through variable‐rate GCD and CV analyses in 10 m ZnCl_2_. Specific capacity reaches 181 mAh g^−1^ at 0.1 A g^−1^, retaining 99 mAh g^−1^ (ca. 55% retention) at 1 A g^−1^ (Figure 3g). Rate‐dependent fading primarily originates from high‐potential plateau degradation according to Figure S25. Sweep‐rate‐dependent CV scanning reveals the anion diffusion kinetic in PDPZ electrodes (Figure S26). The b‐values (0.822 anodic/0.705 cathodic) derived from log(v)‐log(i) plots indicate surface‐controlled kinetics [51]. Quantitative deconvolution shows 54% capacitive contribution at 0.2 mV s^−1^ (Figure 3h), confirming mixed pseudocapacitive/diffusion‐limited anion storage in PDPZ electrodes. The pseudo‐capacitance contribution increases as the scan rate increases. The moderate rate performance and pseudo‐capacitance contribution align with ion diffusion coefficients (*D*_ions_) derived from galvanostatic intermittent titration technique (GITT) [52], which are ca. 10^−10^–10^−11^/10^−11^–10^−12^ cm^2^ s^−1^ for the first/second redox plateau (Figure 3i and Figure S27).

The impressive electrochemical performance of PDPZ in aqueous ZnCl_2_ electrolytes motivated assembly of Cl‐DIBs with Zn anode (Figure S28). The Zn||PDPZ cell can be charged to 1.9 V, delivering high initial discharge capacity of 194 and 236 mAh g^−1^ (based on PDPZ mass) at 0.1 A g^−1^ in 10 and 30 m ZnCl_2_, respectively. Two distinct discharge stages are resolved, particularly in 30 m ZnCl_2_, featuring a high‐voltage plateau at ca. 1.1–1.6 V. The high‐voltage plateau endows the aqueous Cl‐DIB with remarkable energy densities of 175 Wh kg^−1^. The Zn||PDPZ cells exhibit rate capability on par with reported Cl‐DIBs employing highly concentrated ZnCl_2_ electrolytes [53, 54], sustaining discharge capacities of ∼100 mAh g^−1^ at 1 A g^−1^ in both electrolytes. Furthermore, they demonstrate good calendar life, retaining capacities above 100 mAh g^−1^ after 198 h (100 cycles at 0.2 A g^−1^). In 10 m ZnCl_2_ at a high current density of 1.0 A g^−1^, a reversible capacity of 74 mAh g^−1^ is maintained after 2000 cycles. The full‐cell configuration shows compromised cycling stability relative to the three‐electrode system, primarily due to Zn anode degradation. Collectively, as compared in Figures S29 and S30 and Table S5, the Zn||PDPZ Cl‐DIBs achieve comprehensive electrochemical performance that surpasses conventional superchloride counterparts and rivals the forefront of polyatomic‐anion storage in aqueous Zn‐based systems. Notedly, unlike the proposed N^+^‐Cl^−^ bonding in porous polytriphenylamine cathodes [13], the linear conjugated π^+^ framework of PDPZ facilitates efficient electron transfer via extended π‐hole delocalization, enabling rapid kinetics and stable cyclability without recourse to complex porosity engineering.

### 2.4 Dynamic Evolution of Cl^−^‐π^+^ Interactions During Electrochemical Cycling

**FIGURE 4:**
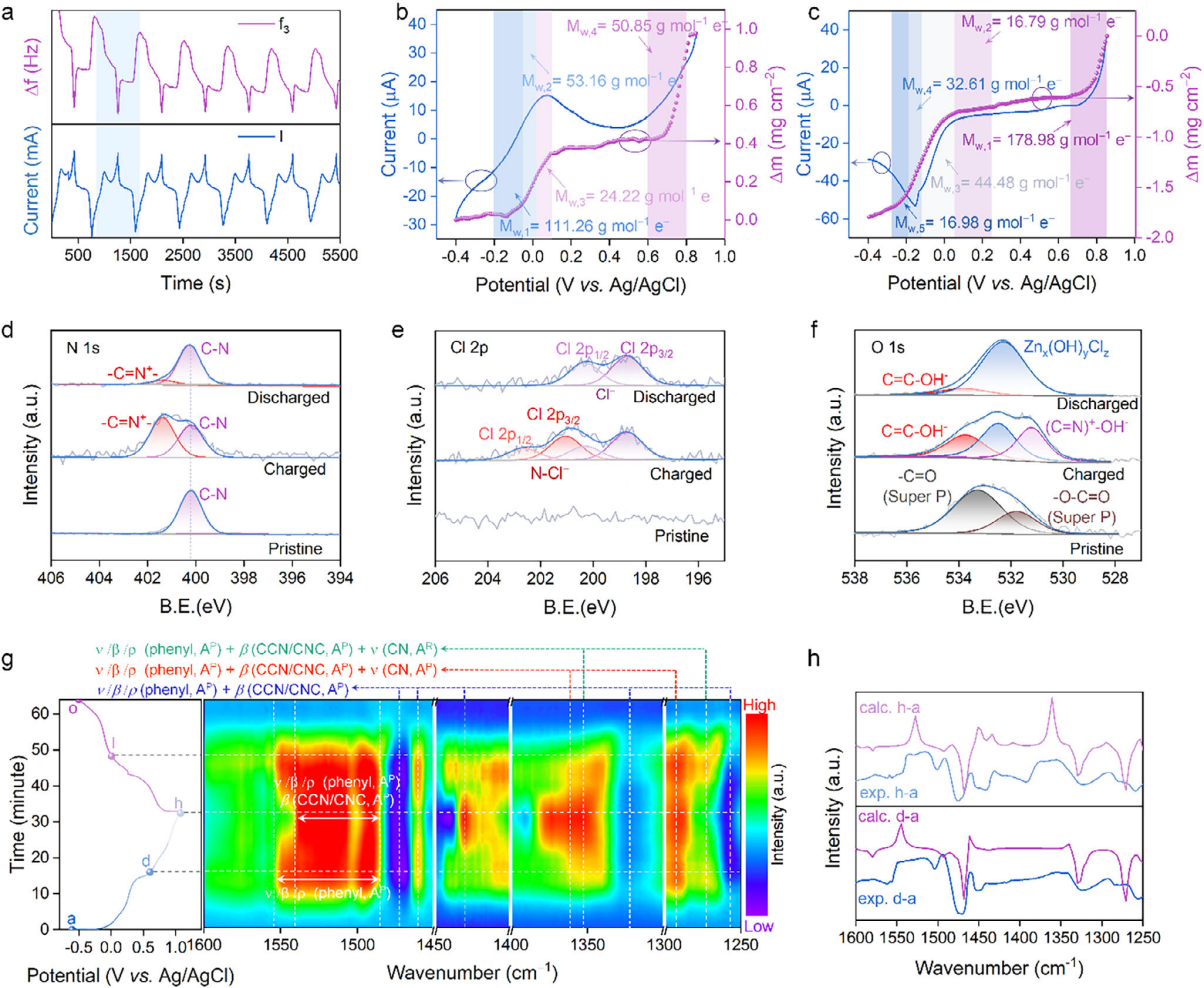
Electrochemically driven structural evolution of PDPZ during anion (de)insertion. (a) Time‐dependent frequency change (*Δf*) of PDPZ during CV. Potential‐dependent mass changes (*Δm*, dots) and CV curves (line) of PDPZ during (b) cathodic (from 0.85 to −0.4 V vs. Ag/AgCl) and (c) anodic (from −0.4 to 0.85 V vs. Ag/AgCl) scanning. EQCM measurements were conducted in 10 m ZnCl_2_ aqueous electrolyte at 3 mV s^−1^ using PDPZ‐coated quartz crystals. Ex situ XPS spectra of (d) N 1s, (e) Cl 2p, and (f) O 1s of PDPZ electrode at pristine, charged, and discharged states (fifth cycle). (g) In situ FTIR spectra of PDPZ electrode during GCD cycle. XPS and FTIR characterizations were performed at ∼200 mA g^−1^. (h) Comparison of calculated (*calc*.) and experimental (*exp*.) difference spectra at selected (dis)charging states labeled in (g). The simulated frequencies were scaled with a scaling factor of 0.964 [61].

The anion storage mechanism of the PDPZ electrode in aqueous ZnCl_2_ electrolyte is further investigated to uncover the anion‐π^+^ interactions by electrochemical quartz crystal microbalance (EQCM), ex situ x‐ray photoelectron spectroscopy (XPS), and in situ attenuated total reflectance Fourier‐transform infrared spectroscopy (ATR‐FTIR). To avoid the prolonged activation time in 30 m ZnCl_2_, 10 m ZnCl_2_ electrolyte is employed to study the anion storage mechanism of the PDPZ electrode. First of all, to identify the inserted anionic carriers while excluding the interference from anionic zinc complexes (e.g. [ZnCl_4_]^2−^ and Zn(H_2_O)_2_Cl_4_^2−^) during redox [53, 55], the XPS survey spectra (Figure S31), elemental mapping and energy‐dispersive x‐ray spectroscopy (EDS) (Figure S32) of the fully charged PDPZ electrode are carefully examined. These analyses confirm negligible anionic zinc‐complex signatures but distinct Cl^−^ signals, verifying chloride as the dominant charge carrier. The real‐time mass change per mole of electron transferred (MPE) of PDPZ electrode during the CV sweep are in situ tracked with EQCM (Figure 4a–c and Figure S33) [9, 56]. In the oxidation process from −0.4 to 0.85 V vs. Ag/AgCl, the MPE value of PDPZ electrode displayed two stages corresponding to the two‐step, one‐electron‐transfer redox reactions. At the first oxidation plateau (−0.2–0.1 V vs. Ag/AgCl), sequential MPE values of 111.26, 53.16 and 24.22 g mol^−1^ e^‒^ (Δ*m*/*Q* = 1.153, 0.551 and 0.251 mg C^−1^) indicate progressive insertion of hydrated Cl^−^ (from Cl^−^·5H_2_O to Cl^−^·H_2_O) [57] and co‐insertion of OH^−^ from free water. Dominant Cl^−^‐π^+^ interactions suppress OH^−^ insertion, limiting its contribution to ∼7.5% (450 µC cm^−2^) of total charge. Upon reaching aqueous swelling equilibrium, the PDPZ cathode accommodates exclusively mono‐hydrated Cl^−^ during the second redox plateau (0.6–0.8 V vs. Ag/AgCl), as evidenced by the characteristic MPE value of 50.85 g mol^−1^ e^−^. Deintercalation profiling further confirms hydrated Cl^−^ as the dominant charge carrier (Figure 4c and Figure S33b). During reductive scanning from 0.85 to 0.65 V, a substantially elevated MPE value of 178.98 g mol^−1^ e^−^ signifies hydrated Cl^−^ extraction coupled with concurrent dehydration of the polymer matrix. Subsequent scanning reveals Cl^−^·H_2_O and Cl^−^ co‐deintercalation at 0.06–−0.19 V, while bifurcated OH^−^ expulsion occurs at 0.26–0.06 V and < −0.2 V.

To elucidate charge‐carrier coordination in PDPZ, we analyzed cycled electrodes with high‐resolution XPS (Figure 4d–f). The N 1s spectra show reversible changes in the quaternary nitrogen redox centers during cycling. In the fully charged state, a new binding energy (BE) peak appears at 401.4 eV, corresponding to C═N^+^ formation, in contrast to the single C─N peak at 400.2 eV observed in pristine PDPZ [11, 58]. Upon discharge, the C═N^+^ peak diminishes concurrently with the recovery of the C─N signal, indicating reversible redox interconversion between these moieties. The Cl 2p and O 1s spectra indicate that Cl^−^ and OH^−^ anions act as charge carriers coordinating with PDPZ^x+^. Peaks in the Cl 2p spectrum at 201.1 and 202.7 eV are assigned to Cl^−^ anions binding with PDPZ^x+^ (PDPZ^x+^···Cl^−^), while O 1s peaks at 531.2 and 533.7 eV correspond to C═N^+^─OH^−^ and aromatic C═C^+^─OH^−^ groups, respectively. The PDPZ^x+^···Cl^−^ bonding via Cl^−^‐π^+^ interaction is of high reversibility, as the higher‐BE Cl 2p signals vanish upon full discharge. The synchronous evolution of the C═N^+^, Cl^−^ and OH^−^ XPS signals demonstrates the reversible uptake and release of anionic species, including hydrated or non‐hydrated chloride and hydroxide ions, consistent with previous EQCM results. However, a residual C═C^+^─OH^−^ signal persists in the discharged state, indicating lower cycling reversibility for OH^−^ species compared to PDPZ^x+^···Cl^−^. Furthermore, the irreversible deposition of Zn_x_(OH)_y_Cl_z_ species on the surface of the cycled PDPZ electrode at the fully discharged state is indicated by combined evidence from EDS mapping (Figure S34), high‐resolution XPS fitting (including Cl 2p, O 1s, Zn 2p/3p core levels and Zn LMM Auger spectrum, Figures S35–S37 and Tables S6 and S7), and Wagner (chemical state) plot analysis (Figure S37c) [59, 60].

To evaluate the influence of Cl^−^‐π^+^ interactions on vibrational frequencies, the calculated IR spectra of d‐DPZ^x+^·xCl^−^ complexes (Figure S38) were compared with those of isolated d‐DPZ^x+^ cations. The comparison of theoretical spectra reveals a blueshift (∼3–5 cm^−1^) for CCN/CNC bending modes and a redshift (11 cm^−1^) for the aromatic C─H out‐of‐plane bending (767 cm^−1^ for d‐DPZ^4+^·4Cl^−^), indicating that Cl^−^‐π^+^ interactions strengthen the polarity of the aromatic skeleton bonds while weakening peripheral C─H bonds.

Time‐dependent ATR‐FTIR spectral evolution of the PDPZ electrode during GCD cycle (Figure 4g and Figures S38 and S39) reveals both the high reversibility of anion insertion/extraction and characteristic vibrational changes during redox processes. The evolution of the experimental vibrational bands aligns with the theoretically predicted trend in spectral frequencies across the oxidation series d‐DPZ→d‐DPZ^2+^·2Cl^−^→d‐DPZ^4+^·4Cl^−^ (Figure 4h and Figure S40), where detailed vibrational assignments (1700–1100 cm^−^^1^) are provided in Figures S41–S44 and Table S8. Phenazine CCN/CNC bending vibrations progressively attenuate during successive oxidation steps, with intensity loss at 1472, 1322, and 1257 cm^−1^. During the first oxidation step (spectra a→d), vibrations of phenyl rings in phenazine moieties ((*v*/*β*/*ρ* (phenyl, A^P^), 1555–1485 cm^−1^) and combined CCN/CNC bending modes of pyrazine rings (*v*/*β*/*ρ* (phenyl, A^P^) + *β* (CCN/CNC, A^P^), centered at 1460, 1353 and 1273 cm^−1^) intensify progressively, confirming anion‐π^+^ interactions with the charged phenazine units. The dramatically increase intensity at 749 cm^−1^ is assigned to C─H out‐of‐plane bending of partially oxidated phenazine moieties (Figure S40). As potential rises to the second oxidation plateau (spectra d→h), the broad band at 1555–1485 cm^−1^ narrows to 1541–1485 cm^−1^, incorporating additional *β* (CCN/CNC, A^P^) and *v* (CN, A^P^) modes in the fully charged state. Concurrently, the vibration band centered at 1353 cm^−1^ shifts progressively to 1362 cm^−1^, while the band at 1460 cm^−1^ diminishes with the emergence and growth of a new band at 1431 cm^−1^. Displacement of Cl^−^ from the pyrazine centroid in the fully charged state may enhance asymmetric vibrations, yielding a more refined spectrum. Furthermore, the sequential appearance and disappearance of weak vibrational bands corresponding to O─H stretches (free water at ∼3750 cm^−1^, binding water of hydrated Cl^−^ at 3200 cm^−1^, and OH^−^ species at 2850 cm^−1^) and N─O stretching (C═N^+^─OH^−^ at 1018 cm^−1^) during successive oxidation steps suggests co‐intercalation of hydrated Cl^−^ and OH^−^ species, respectively. Nevertheless, Cl^−^ and hydrated Cl^−^ govern the PDPZ redox process, evidenced by key aromatic skeletal vibrations and enhanced cycling stability at higher electrolyte concentrations. This dominance arises from a strong Cl^−^‐π^+^ interaction, which effectively inhibits OH^−^ intercalation originating from water dissociation.

**FIGURE 5:**
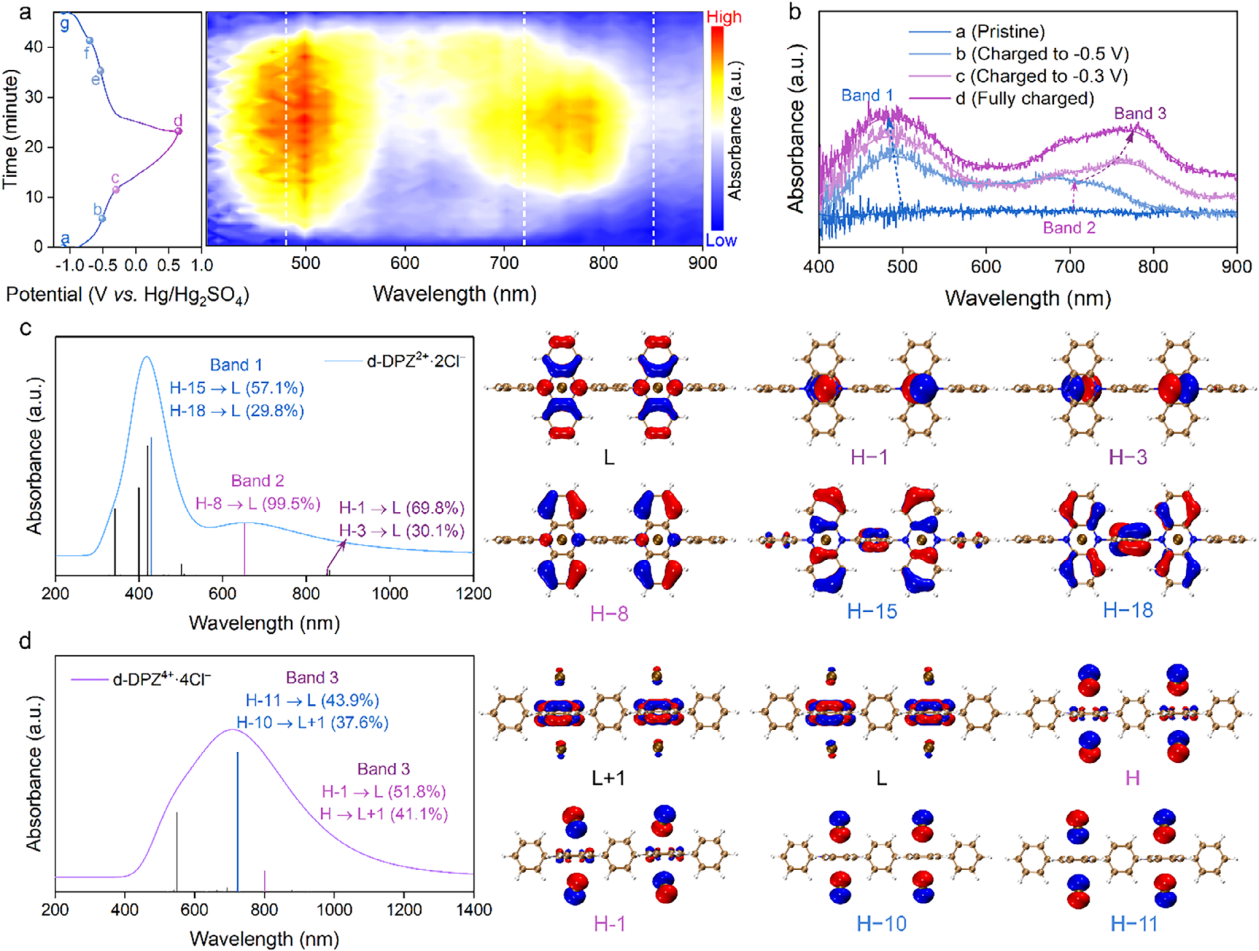
Mechanistical elucidation of ICT between charged PDPZ and Cl^−^. (a) In situ UV–vis–NIR absorbance spectra of PDPZ electrode during galvanostatic cycling. (b) Selected spectra at marked redox states from (a). TD‐DFT calculated UV–vis–NIR absorption profiles with representative frontier molecular orbital distributions: (c) d‐DPZ^2+^·2Cl^‒^ and (d) d‐DPZ^4+^·4Cl^‒^. H and L represent HOMO and LUMO, respectively.

The electronic structure evolution of PDPZ during Cl^−^ insertion/extraction was monitored using in situ UV–vis–NIR spectroscopy (Figure 5a,b). Absorption bands assignments were supported by time‐dependent density functional theory (TD‐DFT) calculations (Figure 5c,d and Table S9). Upon oxidation to −0.5 V vs. Hg/Hg_2_SO_4_ (spectra a→b), characteristic absorption bands centered at ∼490 nm (band 1) and ∼700 nm (band 2) emerge, consistent with the calculated formation of the radical dimer d‐DPZ^2+^·2Cl^−^ (Figure S45a). As illustrated in Figure 5c, band 1 is ascribed to an electronic transition from deep‐lying orbitals (HOMO−15 and HOMO−18) to the LUMO (*f* = 0.1446) of d‐DPZ^2+^·2Cl^−^, occurring between its linked phenyl and phenazine moieties. Whereas, the broad band 2 is mainly attributed to the orbital transition localized at phenazine moieties of d‐DPZ^2+^·2Cl^−^ (HOMO−8→LUMO, *f* = 0.0542). Subsequent oxidation (spectra b→d) induces a slight blueshift of band 1 to ∼480 nm, while a new low‐energy band (band 3) emerges at 720–850 nm. Intramolecular charge transfer (ICT) from Cl^−^ to phenazine moieties in d‐DPZ^4+^·4Cl^−^ complex (HOMO−11→LUMO and HOMO−10→LUMO+1, *f* = 0.1775) dominates the absorption band 3 (Figure 5d). A weak ICT transition with low oscillator strength (*f* = 0.0064) is also observed in d‐DPZ^2+^·2Cl^−^ (*λ* = 850.08 nm, HOMO−1/HOMO−3→LUMO), consistent with Cl^−^ participates in orbital interactions (Figure 2). Calculated spectra comparison of chloride‐coordinated complexes with isolated d‐DPZ^x+^ cations and polyatomic anion complexes (Figures S45b, S46, S47 and Table S9), along with comparative experimental analysis of PDPZ across varied electrolytes (Figures S48b and S49), collaboratively confirms that the near‐infrared absorption in in situ UV–vis–NIR spectra of PDPZ electrodes in aqueous ZnCl_2_ links explicitly to Cl^−^→π^+^ ICT within PDPZ^x+^·xCl^−^. All the optical signals recover to the initial state during the discharge process (Figure S48a), suggesting an excellent reversible redox process. Consequently, these spectroelectrochemical results highlight the ionic‐covalent character of the bonding between Cl^−^ and charged PDPZ frameworks, playing a fundamental role in facilitating state‐of‐the‐art Cl^−^ storage.

## 3 Conclusion

In summary, we demonstrate the hybrid ionic‐covalent Cl^−^‐π^+^ interaction between the strongly nucleophilic Cl^−^ and poly(arylamine) hosts involves both electrostatic interactions and orbital contributions. DFT analyses reveal that enhanced π‐delocalization in phenazine‐integrated PDPZ^x+^ frameworks (relative to PTPA^x+^) stabilizes the π^+^ charge while maintaining the partial covalent character of Cl^−^‐π^+^ interactions in PDPZ ^x+^·xCl^−^ complexes through orbital‐mediated Cl^−^→π^+^ charge transfer. The Zn||PDPZ cell achieves a high anion storage capacity of 236 mAh g^−1^ and energy density of 175 Wh kg^−1^ (30 m ZnCl_2_), with long calendar life as Cl‐DIB cathodes, outperforming conventional superchloride systems and rivaling state‐of‐the‐art polyatomic‐anion storage in aqueous Zn‐based devices. In situ ATR‐FTIR spectroscopy visualizes the dynamic skeletal vibrations of PDPZ during electrochemical Cl^−^ insertion/extraction, demonstrating the contribution of the entire π^+^ framework to Cl^−^–π^+^ interactions. Furthermore, in situ absorption spectra confirm charge transfer from Cl^−^ to the phenazine moieties of the cationic PDPZ, elucidating the orbital nature of the Cl^−^–π^+^ binding mechanism. These fundamental insights into Cl^−^‐π^+^ interaction‐driven anion storage extend beyond pure electrostatic attraction, offering critical guidance for designing next‐generation high‐performance organic cathodes and advancing both fundamental understanding and practical applications in anion coordination chemistry.

## Author Contributions

Z.L. and W.H. conceived the idea and supervised the project. Z.L. and J.W. designed the experiments. T.S. carried out materials synthesis and characterizations with the assistance of Y.Z. and Z.Z. T.S. fabricated and characterized electrodes with the assistance of J.W., Q.X., and Y.Z. T.S., and J.W. conducted DFT calculation and analyse the interaction between PAAs and anions. T.S., J.W., and Z.L. conducted the analysis of in situ FTIR and UV–vis–NIR spectra. J.W. and T.S. cowrote the first draft of the manuscript. Z.L. and W.H. provided major revisions. All authors contributed to analyse the data and discussed the results.

## Conflicts of Interest

The authors declare no conflicts of interest.

## Supporting information




**Supporting File**: advs75896‐sup‐0001‐SuppMat.docx.

## Data Availability

The data that support the findings of this study are available from the corresponding author upon reasonable request.
