# Supplementary material for: Beyond Electrostatics: Anion‐π+ Orbital Hybridization Underpins High‐Performance Chloride Storage in Poly(arylamine) Organic Cathodes
Source: Adv Sci (Weinh). 2026 Jun 3;13(48):e75896. doi: 10.1002/advs.75896 (PMC13336400; doi:10.1002/advs.75896)
Supplement: Supplementary file 1 [file ADVS-13-e75896-s001.docx]

Supporting Information

**Beyond Electrostatics: Anion-π^+^ Orbital Hybridization Underpins High-Performance Chloride Storage in Poly(arylamine) Organic Cathodes**

Tiantian She^1†^, Jiena Weng^1†^*, Jie Wang^1^, Qiao Xi^1^, Yangyang Zhang^1^, Zijie Zhang^1^, Zongqiong Lin^1^* & Wei Huang^1,2,3^*

^1^ State Key Laboratory of Flexible Electronics (LOFE) & Institute of Flexible Electronics (IFE), Shaanxi Key Laboratory of Flexible Electronics, MIIT Key Laboratory of Flexible Electronics (KLoFE), Northwestern Polytechnical University, Xi'an 710072, China

^2^ State Key Laboratory of Organic Electronics and Information Displays & Institute of Advanced Materials (IAM), Nanjing University of Posts & Telecommunications, Nanjing 210023, China

^3^ Key Laboratory of Flexible Electronics & Institute of Advanced Materials, Nanjing Tech University, Nanjing 211816, China

*Corresponding Author(s): iamjnweng@nwpu.edu.cn; iamzqlin@nwpu.edu.cn; vc@nwpu.edu.cn;

^†^ T. She and J. Weng contributed equally to this work.

# **Table of Contents**

[**Table of Contents**	S1](#_Toc221992723)

[**Supplemental Methods**	S2](#_Toc221992724)

[**1.**	**Experimental Section**	S2](#_Toc221992725)

[**2.**	**Computational Methods**	S4](#_Toc221992726)

[**3.**	**Structures and Characterization of Poly(arylamine)s (PAAs)**	S7](#_Toc221992727)

[**4.**	**Electronic Structures and Anion-π^+^ Interactions of PAAs and Corresponding Ion Pairs**	S11](#_Toc221992728)

[**5.**	**Comparative Cl^−^ Storage in PAA-Based Electrodes**	S24](#_Toc221992729)

[**6.**	**Cl^−^ Storage Mechanism of PDPZ**	S32](#_Toc221992730)

[**7.**	**Theoretical Calculations and Experimental in-situ ATR-FTIR/** **UV-vis-NIR Spectra**	S37](#_Toc221992731)

[**8.**	**Supporting Tables**	S46](#_Toc221992732)

[**9.**	**References**	S56](#_Toc221992733)

# **Supplemental Methods**

# 1. **Experimental Section**

1.1 Materials

Triphenylamine (TPA, 98%), sodium tert-butoxide (99%), and 2-dicyclohexylphosphino-2’,4’,6’-triisopropylbiphenyl (XPhos, 98%) were purchased from Shanghai Titan Scientific Co., Ltd. Ferric chloride (FeCl_3_, 99.9%) and palladium acetate (99.9%) were obtained from Sigma-Aldrich (Shanghai) Trading Co., Ltd. 5,10-dihydrophenazine (95%) was purchased from Accela ChemBio Co., Ltd. 1,4-dibromobenzene (99%) and polyvinylidene fluoride (PVDF) were purchased from Shanghai Macklin Biochemical Co., Ltd. Zinc chloride (ZnCl_2_, 98%) was purchased from Thermo Fisher Scientific (China) Co., Ltd. Zinc trifluoromethanesulfonate (Zn(OTf)_2_, 98%) and Zinc bis(trifluoromethanesulfonyl) imide (Zn(TFSI)_2_, 98%) were purchased from TCI (Shanghai) Chemical Trading Co., Ltd. Lithium bis(trifluoromethanesulfonyl) imide (LiTFSI, 99%) was purchased from J&K Scientific Co., Ltd. Sodium perchlorate (NaClO_4_, 99.99%) and Super P were obtained from Shanghai Aladdin Biochemical Technology Co., Ltd. All chemicals are commercially available and used without further purification.

1.2 Synthesis of PTPA and PDPZ

PTPA was synthesized following a modified literature procedure.^1,2^ Briefly, TPA (3.06 g, 12.5 mmol) was dissolved in chloroform (50 mL) under ambient conditions. Ferric FeCl_3_ (2.02 g, 12.5 mmol) was added portionwise (4 times) at 1 h intervals. The reaction mixture was stirred at room temperature for 4 h to complete the polymerization. The resulting solution was then precipitated into methanol (100 mL), yielding a light green powder, which was collected by filtration and washed with methanol (3 × 50 mL). Further purification was achieved by re-precipitation from an acetone/5% aqueous ammonia solution. The final yellow solid product was dried under vacuum at 85 °C for 12 h. MALDI-TOF Mass: C_72_H_54_N_4_, Exact Mass: 974.43, Found: 974.335 (n≈4); C_90_H_67_N_5_, Exact Mass: 1217.54, Found: 1217.459 (n≈5); C_108_H_80_N_6_, Exact Mass: 1460.64, Found: 1460.581 (n≈6); C_270_H_197_N_15_, Exact Mass: 3648.59, Found: 3648.673 (n≈15). FTIR (KBr): 1594, 1490, 1325, 1274, 1176, 818 cm^−1^.

**Scheme S1.** Synthetic route for PTPA.

PDPZ was synthesized according to modified literature procedures.^3^ In a typical procedure, a Schlenk flask was charged with 5,10-dihydrophenazine (408 mg, 2.2 mmol), 1,4-dibromobenzene (472 mg, 2 mmol), palladium acetate (29 mg, 0.13 mmol), XPhos (124 mg, 0.26 mmol), sodium tert-butoxide (577 mg, 6 mmol), and anhydrous *o*-xylene (30 mL). The system was degassed via three freeze-pump-thaw cycles and subsequently purged with argon under vigorous stirring for 15 min. The mixture was heated at 125 °C for 24 h, followed by 155 °C for an additional 48 h under argon atmosphere. After cooling to ambient temperature, the crude product was collected by filtration and sequentially washed with copious amounts of hot *o*-xylene, methanol, and deionized water. Further purification was achieved by Soxhlet extraction using dichloromethane, tetrahydrofuran, methanol, and water, respectively. The resulting brownish-black solid was dried under vacuum at 90 °C overnight to afford the pure product. MALDI-TOF Mass: C_42_H_30_N_4_, Exact Mass: 590.25, Found: 590.484 (n≈2); C_60_H_42_N_6_, Exact Mass: 846.35, Found: 846.697 (n≈3); C_72_H_50_N_8_, Exact Mass: 1026.42, Found: 1025.844 (n≈4); C_90_H_62_N_10_, Exact Mass: 1282.52, Found: 1282.069 (n≈10); Elemental Analysis: Found: C, 78.04; H, 4.48; N, 9.27. ^13^C MAS NMR (400 MHz): 135.7, 121.4, 113.2 ppm. FTIR (KBr): 1600, 1484, 1280, 733 cm^−1^.

**Scheme S2.** Synthetic route for PDPZ.

1.3 Material Characterizations

Matrix-assisted laser desorption ionization-time of flight (MALDI-TOF) mass spectrometry was performed using an AB Sciex 5800 MALDI-TOF/TOF spectrometer (USA). Elemental analysis was carried out on a Vario EL Cube analyzer (Elementar, Germany). Solid-state ^13^C magic-angle spinning nuclear magnetic resonance (^13^C MAS NMR) spectra were recorded at 400 MHz on an AVANCE III HD spectrometer (Bruker, Germany).

The morphology, elemental mapping and energy-dispersive X-ray spectroscopy (EDS) analyses were conducted on a ZEISS Gemini SEM 300 (Germany) coupled with a Bruker Nano GmbH (Am Studio 2D, Germany) at an accelerating voltage of 15 kV. X-ray photoelectron spectroscopy (XPS) measurements were performed using an Escalab 250Xi system (Thermo Fisher Scientific, USA) at the Northwest Institute for Non-ferrous Metal Research (China), with binding energies calibrated against the C 1s peak (C‒C bond) at 284.8 eV. All electrode samples subjected to ex-situ characterization (XPS and elemental mapping/EDS) were harvested from Swagelok cells under specific state-of-charge (SOC) conditions. Prior to analysis, the electrodes underwent five successive rinses with distilled water to thoroughly remove residual electrolyte, followed by vacuum-drying at 60 °C for 24 h. Processed electrodes were individually sealed in argon-filled containers within the glove box (H_2_O/O_2_ < 0.1 ppm) and stored until prior to testing.

In-situ attenuated total reflectance Fourier-transform infrared spectroscopy (ATR-FTIR) spectra were acquired on a Nicolet iS50 spectrometer (Thermo Scientific, USA) equipped with a diamond ATR accessory (Linglu Instruments, China), collecting 32 scans per spectrum at 4 cm⁻^1^ resolution. In-situ UV-vis-NIR reflectance spectra were monitored using a QEpro UV-Vis spectrometer (Ocean Optics, USA). Both aforementioned in situ electrochemical characterizations were conducted in a three-electrode system, with charge/discharge processes synchronously controlled by a LAND-CT2001A battery tester (Wuhan LAND Electronics, China). EQCM measurements employed Au-coated quartz crystal sensors and a QSense Analyzer (Biolin Scientific) coupled with an electrochemical workstation (Autolab PGSTAT302N, Metrohm). A PDPZ thin film was spin-coated onto the Au sensor from 5 mg/mL trifluoroacetic acid/NMP (~1:10 v/v) solution at 1500 rpm for 120 s, then vacuum-dried at 110 °C for 3 h. A three-electrode cell was adopted for EQCM measurements, in which the PDPZ-modified Au sensor, Ag/AgCl, and platinum wire served as working electrode, reference electrode and counter electrode, respectively. After establishing the fundamental resonance frequency baseline, 10 m ZnCl_2_ aqueous electrolyte was injected into the fluid cell. The system stabilized for 15 min before frequency monitoring. In situ mass changes during charge/discharge were measured simultaneously with cyclic voltammetry (3 mV s^‒1^), with the second stabilized cycle used for analysis.

1.4 Electrode Fabrication and Electrochemical Measurements

PAA electrodes were fabricated by mixing the as-synthesized PAA, Super P and PVDF with a mass ratio of 4:4:2 into NMP solvent. The resulting slurry was blade-coated onto graphite foil (20 μm) and vacuum-dried at 110 °C for 24 h. Electrodes (10 mm diameter) exhibited active material loadings of 0.8‒1 mg cm^−2^. Electrochemical testing employed custom-modified Swagelok cells with a three-electrode configuration, assembling with PAA-based working electrodes, over-capacitive activated carbon counter electrode and Hg/Hg_2_SO_4_ (in saturated K_2_SO_4_) reference electrode. Both PTPA and PDPZ electrodes are investigated in aqueous ZnCl_2_ electrolytes with different concentration (3 m, 10 m, and 30 m, respectively). For PTPA electrodes, more aqueous electrolytes were employed, including 3 m Zn(OTf)_2_, 10 m LiTFSI + 1 m Zn(TFSI)_2_, and 17 m NaClO_4_ + 0.5 m Zn(OTf)_2_. Two-electrode Zn||PDPZ cells were assembled with PDPZ as the cathode, Zn metal as the anode, glass fiber as separator, and aqueous ZnCl_2_ (10 m, 100 µL) as electrolyte. Galvanostatic charge/discharge (GCD) profiles and cycling stability were evaluated using a LAND CT2001A battery test system. Cyclic voltammetry (CV) was performed on an Autolab PGSTAT302N potentiostat (Metrohm).

# 2. **Computational Methods**

2.1 Density Functional Theory Calculations

All DFT calculations were performed using the Gaussian 16 software package^4^ using B3LYP hybrid functional.^5^ Geometric optimizations and vibrational frequency analyses for neutral d-TPA/d-DPZ molecules and their ionic species were carried out with 6-31G(d) basis sets.^6^ For coordination complexes involving positively charged d-TPA/d-DPZ moieties and Cl⁻ counterions, geometry optimizations employed 6-31+G(d) basis sets^7,8^ with Grimme's D3 dispersion correction (GD3BJ) for describing non-covalent interactions.^9,10^ The single-point energy was further calculated at 6-311+G (d) basis sets.^11^ To account for the solvation effect of water, the polarizable continuum model solvation model (PCM) was chosen.^12^

Molecular orbitals (MO), interaction region indicator (IRI), electrostatic potential (ESP) distributions, atomic dipole corrected Hirshfeld (ADCH) atomic charge analysis, and extended transition state-natural orbitals for chemical valence (ETS-NOCV) were analyzed using Multiwfn 3.8^13^ and visualized with the Visual Molecular Dynamics (VMD) package.^14^ The isosurface maps of electron density difference (EDD) were visualized by the Visualization for Electronic and Structural Analysis (VESTA, ver. 3. 90. 0a) software.^15^ Aromaticity was evaluated via nucleus-independent chemical shift (NICS) indices computed using the gauge-including atomic orbital (GIAO) method.^16^ The geometric centers of aromatic rings were algorithmically determined in Multiwfn to compute the NICS(1)ZZ component, which selectively reflects π-electron contributions.^17^ Current density topology was characterized through anisotropy of the induced current density (ACID) simulations, following Herges' implementation to map ring currents and magnetic shielding effects.^18^

2.2 Calculated of Gibbs free energy change (*ΔG*).

*The Gibbs free energy change (ΔG)* of the PAAs at each step of the reaction process can be calculated using the following formula:^19^

$$\Delta G=G_{f}-G_{i}-{nG}_{anion}$$

(Eq. S1)

where G_f_, G_i_, and G_anion_ are the Gibbs free energy for the final state of PAAs, the initial state of PAAs, and the anions (Cl^−^, OTf^−^ and TFSI^−^), respectively; n represents the number of anions.

2.3 Interactions Simulation.

*Energy decomposition analysis (EDA)* was performed using the sobEDA method. The interaction energy (Δ*E*_int_) is partitioned into four key components and expressed as:

$$\Delta E_{int}=\Delta E_{els}+\Delta E_{xrep}+\Delta E_{orb}+\Delta E_{c}$$

(Eq. S2)

the interaction energy was partitioned into four key components: Δ*E*_els_, electrostatic interaction energy; Δ*E*_xrep_, exchange-repulsion energy, derived from the combination of exchange energy (Δ*E*_x_) and Pauli repulsion (Δ*E*_rep_); Δ*E*_orb_, orbital interaction energy; and Δ*E*_c_, coulomb correlation term, incorporating both DFT correlation energy (Δ*E*_DFTc_) and dispersion correction (Δ*E*_dc_).

2.4 Redox Electron Transfer Number

The theoretical capacity (*C_m_*, mAh g^−1^) of an organic cathode was determined based on the following form:

$$C_{m}=\frac{n\times F}{3.6\times M}$$

(Eq. S3)

where F is the Faraday constant (96485 C mol^−1^), M is the molecular weight of an organic material (g mol^−1^). For the PDPZ electrode, the theoretical capacity was calculated as 209 mAh g^−1^ based on its repeating unit molecular weight of 256.3 g mol^−1^ and two-electron transfer per repeating redox unit. Under the same calculation framework, PTPA demonstrated a theoretical capacity of 110 mAh g^−1^.

2.5 Capacitive Contribution

The charge storage kinetics of PDPZ electrode were investigated using the following relationship:^20^

$$i=kv^{b}$$

(Eq. S4)

where *k* and *b* are empirical constants, while *i* and *v* denote current density and scan rate, respectively. A *b*-value approaching 0.5 indicates a diffusion-controlled mechanism, whereas *b* = 1.0 corresponds to a surface-dominated redox process.

The quantitative separation of capacitive and diffusion contributions can be calculated through the equation:

$$i=k_{1}v+k_{2}v^{1/2}$$

(Eq. S5)

where *k_1_* and *k_2_* represent the proportionality constants for capacitive and diffusion-controlled components, respectively. Dividing both sides by *v^1/2^* , the expression transforms into:^21^

$$i/v^{1/2}=k_{1}v^{1/2}+k_{2}$$

(Eq. S6)

A linear correlation between *i/v^1/2^* and *v^1/2^* enables quantitative deconvolution of capacitive contributions through linear regression analysis. The slope and intercept correspond to the kinetic coefficients *k_1_* (capacitive) and *k_2_* (diffusion-controlled), respectively. By systematically applying this method across varying potentials and scan rates, the relative capacities of surface-controlled and diffusion-controlled mechanisms can be precisely quantified.

2.6 Diffusivity measurement

Galvanostatic intermittent titration technique (GITT) measurement was performed on a Swagelok cell with a PDPZ working electrode, over-capacitive activated carbon counter electrode, and Hg/Hg_2_SO_4_ (in saturated K_2_SO_4_) reference electrodes. Prior to the measurement, the cell was fully charged and discharged at the current densities of 0.2 A g^−1^. The GITT current input consisted of 15 charge pulses. Each pulse lasts 3 minutes followed by 30 minutes relaxation. The solid phase diffusivity of Cl^−^ in PDPZ was calculated using the GITT diffusivity formula:

$$D=\frac{4}{\pi\tau}\left( \frac{n_{M}V_{M}}{S} \right)^{2}\left( \frac{{\Delta V}_{S}}{{\Delta V}_{t}} \right)^{2}$$

(Eq. S7)

where *n_M_* and *V_M_* are the mass (mol) and molar volume (cm^3^·mol^–1^) of the active material, respectively, S is the interfacial area of the active material (cm^2^), *τ* is the time duration of the pulse (s), *ΔV*_s_ is the difference between the steady-state potentials before and after a current pulse and thus indicates the change in the potential due to thermodynamics, and *ΔV*_t_ is the magnitude of the potential changes (without the IR drop) during the current pulse.

2.7 Modelling of EQCM Data

In general, the frequency change of the quartz resonator (*Δf*, Hz) is converted into a mass change (*Δm*, ng cm^−2^) of the electrode coated on the quartz crystal by the Sauerbrey’s equation:^22,23^

$$\Delta m=-C_{f}\times\Delta f$$

(Eq. S8)

where the constant *C_f_* (17.7 ng cm^−2^·Hz^−1^ for 5 MHz crystals) is the sensitivity factor of the crystal, *Δf* is the quartz resonance frequency (Hz).

The absolute theoretical *Δm*/Q value was calculated based on Faraday’s law as follows:

$$\frac{\Delta m}{Q}=\frac{M}{F\times n}$$

(Eq. S9)

where *M* is molar weight of the intercalated ions (g mol^−1^), Q is the charge quantity (C cm^−2^), F is the Faraday constant (96485 C mol^−1^), and n is the number of electrons transferred per ion.

# 3. **Structures and Characterization of Poly(arylamine)s (PAAs)**

**Figure S1.** (a) Schematic diagrams of the orbital structure of triphenylamine (TPA) and 5,10-diphenyl-5,10-dihydrophenazine (DPZ); (b) Dimer models adopted for DFT calculation, denoted as d-TPA (left panel) and d-DPZ (right panel), respectively.

**Figure S2.** FTIR spectra of the as-synthesized PAAs and their corresponding monomers. (a) Comparison of FTIR spectra of PTPA with TPA monomer and (b) PDPZ with precursor monomers1,4-dibromobenzene and 5,10-dihydrophenazine.

**Note to Figure S2.** The FTIR analysis demonstrates that the prominent absorption band at 816 cm^−1^ in PTPA can be assigned to the characteristic C–H out-of-plane bending vibration of 1,4-disubstituted benzene rings, which aligns with previously reported spectral features for successfully synthesized PTPA.^1,2,24^ Notably, the disappearance of two diagnostic vibration bands provides critical evidence for polymerization completion: the N–H stretching vibration at 3252 cm^−1^ (originating from 5,10-dihydrophenazine) and the C–Br stretching vibration at 468 cm^−1^ (characteristic of 1,4-dibromobenzene). These changes confirm the effective consumption of starting materials and successful formation of PDPZ through the polymerization process.^25^

**Figure S3.** Solid-state ^13^C NMR spectrum of aromatic region of PDPZ.

**Figure S4.** Matrix-assisted laser desorption/ionization time-of-flight (MALDI-TOF) mass spectrum of PTPA and corresponding fragmentations.

**Figure S5**. MALDI-TOF mass spectrum of PDPZ and corresponding fragmentations.

**Note to Figure S4** and **S5.** MALDI-TOF mass spectrometry analysis demonstrated a periodic increment in molecular weights (m/z) for both polymers. The analysis identified oligomers with polymerization degrees of 6 (and/or higher) for PTPA (**Figure S4**) and 5 (and/or higher) for PDPZ (**Figure S5**), respectively.

# 4. **Electronic Structures and Anion-π^+^ Interactions of PAAs and Corresponding Ion Pairs**

**Figure S6.** Optimized structures, molecular orbitals (MOs) distributions, energy levels and energy gaps of neutral d-TPA and d-DPZ. HOMO and LUMO denote the highest occupied molecular orbital and lowest unoccupied molecular orbital, respectively.

**Figure S7.** NICS(1)zz values of positively charged d-TPA/d-DPZ coordinated with/without Cl^−^ anions binding.

**Figure S8.** ACID plots of neutral and charged d-TPA.

**Figure S9.** (a) Molecular electrostatic potential (ESP) mappings of d-DPZ and its chlorinated derivatives (d-DPZ^x+^·xCl⁻), demonstrating structural evolution through different chloride reaction pathways and (b) corresponding calculated Gibbs free energy changes per electron unit (*ΔG/e)* of possible reaction paths.

**Note to Figure S9.** The Gibbs free energy change per electron (ΔG/*e*) is calculated to evaluate the redox reaction sequences of d-TPA and d-DPZ with Cl⁻ (**Figure S9** and **Table S1**). The more favorable ΔG/*e* value for step 1 compared to step 3 (5.03 eV) excludes the possibility of a concerted four-electron pathway (**Figure S9b**). The greater driving force (less positive ΔG/*e*) for step 1 relative to step 2 further supports the sequential nature of the two-electron oxidation process.^26^

**Figure S10.** Interaction region indicator (IRI = 1.0) analysis of d-TPA and d-TPA^2+^·2Cl^−^.

**Figure S11.** IRI analysis of d-DPZ, d-DPZ^2+^·2Cl^−^, and d-DPZ^4+^·4Cl^−^.

**Note to** **Figure S10–S11.** Interaction Region Indicator (IRI) analyses^27^ were conducted using the Multiwfn software, with isosurface visualization and scatter plots generated via the VMD program. In this methodology, the sign(*λ*₂)*ρ* function was employed to map intermolecular interaction regions through isosurface projections, enabling discrimination between attractive and repulsive interactions. Here, *ρ* denotes the electron density, while sign(*λ*₂) corresponds to the algebraic sign of the second largest eigenvalue (*λ*₂) derived from the electron density Hessian matrix.

The sign(*λ*₂)*ρ* function was plotted within the range of −0.04 to 0.02 a.u. (**Figures S10** and **S11**), with values color-coded as follows: red (positive), green (near-zero), and blue (negative). The interaction nature was quantified by both the magnitude and sign of sign(*λ*₂)*ρ*. Blue regions (sign(λ₂)ρ < 0) correspond to attractive interactions, with color intensity proportional to strength. These encompass hydrogen bonds, strong halogen bonds, and related noncovalent forces. Green regions (sign(*λ*₂)*ρ* ≈ 0) indicate weak van der Waals (*vdW*) interactions. Red regions (sign(*λ*₂)*ρ* > 0) represent steric repulsion, predominantly observed in cyclic and caged systems, where elevated values denote stronger repulsive effects. Thus, high absolute values of sign(*λ*₂)*ρ* signify strong interactions (attractive or repulsive), while low values reflect weaker interactions.^28^ IRI identifies two distinct isosurface features (sign(λ_2_)ρ = ±0.006 a.u.) in d-TPA^2+^·2Cl^−^, which is absent in neutral d-TPA (**Figure S10**), indicating the presence of weak interaction between d-TPA^2+^ and Cl^−^. In contrast, IRI analysis of d-DPZ^2+^·2Cl^−^ and d-DPZ^4+^·4Cl^−^ reveals interfacial green isosurfaces between Cl^−^ anions and pyrazine rings, confirming anion-π^+^ interactions (**Figure S11**). The most negative sign(λ_2_)ρ value progressively shifts from −0.008 to −0.01 a.u. with increasing oxidation state, indicating enhanced π-electron deficiency and strengthened anion-π^+^ coupling.

**Figure S12.** Optimized structures, HOMO, LUMO and energy gaps of d-TPA^2+^, d-DPZ^2+^, d-DPZ^4+^.

Figure S13. Optimized structures, molecular orbital distributions, energy levels and energy gaps of coordination compounds of d-TPA^2+^·2Cl^−^, d-DPZ^2+^·2Cl^−^, and d-DPZ^4+^·4Cl^−^.

Figure S14. ESP analysis of d-TPA^2+^·2Cl^−^, d-DPZ^2+^·2Cl^−^, and d-DPZ^4+^·4Cl^−^, top view (left panel) and side view (right panel).

**Figure S15.** ETS-NOCV analysis. Two most prominent orbital interactions (*ΔE_orb_*) are revealed by the deformation density plots for d-TPA^2+^·2Cl^−^, d-DPZ^2+^·2Cl^−^, and d-DPZ^4+^·4Cl^−^. The isosurface visualization (isovalue = 0.0015 a.u.) employs a dual-color scheme: green regions indicate electron density accumulation (inflow) while blue regions correspond to electron density depletion (outflow), effectively mapping the charge redistribution patterns in these coordination systems.

**Notes to** **Figure S15:** The ETS-NOCV facilitates quantitative decomposition of Δ*E*_orb_ into distinct orbital contributions while simultaneously visualizing electron redistribution patterns arising from fragment interactions. Through superposition of paired NOCV deformation densities, this approach enables systematic assignment of specific orbital interactions to particular bonding types. Such combined quantitative-visual analysis delivers comprehensive insights into bonding characteristics and inter-fragment electron transfer mechanisms.^29,30^ As demonstrated in **Figure S15**, the cumulative contribution from the first two NOCV pairs (NOCV pair1 + pair2) in d-TPA^2+^·2Cl^−^ predominantly arises from (1) direct Cl^−^···C1/C2 interactions and (2) charge transfer from chloride ions to nitrogen centers. Notably, the non-aromatic pyrazine rings in d-DPZ^2+^ restrict electron transfer exclusively to the Cl^−^→N direction in d-DPZ^2+^·2Cl^−^. In contrast, the fully oxidized d-DPZ^4+^ system exhibits enhanced Cl^−^-π^+^ interaction with the macrocyclic framework, where the global aromaticity and extended π-conjugation of the phenazine ring facilitate effective charge delocalization and π-system participation in Cl⁻ binding.

**Figure S16.** Atomic dipole moment-corrected Hirshfeld (ADCH) atomic charges analysis for d-TPA^2+^·2Cl^−^, d-DPZ^2+^·2Cl^−^, and d-DPZ^4+^·4Cl^−^, respectively.

**Notes to Figure S16:** The atomic charge redistribution during the formation of d-TPA^2+^·2Cl^−^, d-DPZ^2+^·2Cl^−^, and d-DPZ^4+^·4Cl^−^ complexes were visualized through ADCH mapping, with red and blue colors denoting regions of electron depletion (increased positive charge) and accumulation (decreased positive charge), respectively. As illustrated in **Figure S16**, Cl^−^ (shown in red) underwent electron loss, thereby acquiring more positive charges. Conversely, most atoms within the cationic moieties appeared in blue, demonstrating charge reduction (i.e., less positive charge) through electron density accumulation.

**Figure S17.** Electron-density difference (EDD) analysis of PAA^x+^·xCl^–^. The EDD isosurfaces (isovalue = 0.0015 a.u.) illustrate the coordination of d-TPA with 2Cl^−^ (top), d-DPZ with 2Cl^−^ (middle), and d-DPZ with 4Cl^−^ (bottom), displaying in top (left) and side (right) views. Yellow and cyan regions correspond to electron accumulation and depletion, respectively.

**Figure S18.** Superposition of the results of AIM and IGMH analyses onto the optimized structures of both PAA^x+^·xOTf^−^ and PAA^x+^·xTFSI^−^. The bond paths and critical (3, −1) points (from AIM) are shown as orange lines and spheres, and blue-green areas (from IGMH) indicate interactions. Electron density values at these BCPs are provided in **Table S2**.

**Figure S19.** The selected NOCV-pairs deformation densities of PAA^x+^·xOTf^−^ and PAA^x+^·xTFSI^−^. The isosurface visualization (isovalue = 0.0015 a.u.) employs a dual-color scheme: green regions indicate electron density accumulation (inflow) while blue regions correspond to electron density depletion (outflow), effectively mapping the charge redistribution patterns in these coordination systems.

# 5. **Comparative Cl^−^ Storage in PAA-Based Electrodes**

**Figure S20.** (a) Galvanostatic charge-discharge (GCD) profiles and (b) activation behavior of PTPA electrodes during cycle in 30 m ZnCl_2_ at the current densities of 0.2 A g^−1^.

**Notes to Figure S20:** In 10 m ZnCl_2_ electrolyte, PTPA shows obvious capacity fading during the initial 100 cycles. This phenomenon can be mainly attributed to the accelerated accumulation of oxygenated intermediates (e.g., ·OH, ·O_2_^2^⁻) produced by free-water oxidation in the dilute electrolyte. In contrast, in 30 m ZnCl_2_ electrolyte, PTPA undergoes a distinct activation process with a gradual capacity increase. This activation behavior originates from the higher viscosity and lower ionic conductivity of the highly concentrated electrolyte.

**Figure S21.** Specific capacities of (a) PTPA and (b) PDPZ dependence on anion species. GCD profiles of PTPA and PDPZ electrodes under the current densities of 0.2 A g^−1^ in various aqueous zinc-ion electrolytes.

**Figure S22.** GCD profiles (a, b) and Coulombic efficiency (c, d) of PDPZ electrode at the current densities of 0.2 A g^−1^ in 10 m ZnCl_2_ during cycling.

**Figure S23.** Evolution of the electrode interface during cycling. (a) High-resolution Zn 2p_3/2_ XPS spectra at the discharged state after the 5^th^ and 50^th^ cycles. (b) Distribution of relaxation times (DRT) curves obtained at selected discharge cycles (1^st^, 5^th^, 20^th^, and 50^th^). (c) Magnified view of the high-frequency region from (b).

**Note to Figure S22–S23.** The capacity fading of PDPZ electrode in 10 m ZnCl_2_ (aqueous) proceeds primarily in two distinct stages (**Figure S22**). The irreversible formation of Zn_x_(OH)_y_Cl_z_ (served as solid–electrolyte interphase (SEI) on PDPZ electrode) and its dynamic evolution dominating the initial stage, while intrinsic side reactions of the active material govern the long-term cycling stage. Firstly, the GCD profiles show a charge capacity of 297 mAh g⁻¹ (approximately 1.4 times the theoretical specific capacity) in the first cycle, followed by a capacity loss of ~110 mAh g^−1^ (37%) during the first discharge, resulting in a low initial Coulombic efficiency (CE) of ~63% (**Figure S22a** and **S22c**). This significant initial capacity loss is attributed to the irreversible oxidation of free water in the electrolyte at elevated potentials, which generates oxygen-containing intermediates (e.g., ·OH, ·O_2_^2−^); these intermediates further participate in the irreversible deposition of Zn_x_(OH)_y_Cl_z_ on the electrode surface, consistent with the reaction mechanism reported in previous literature.^31^ Furthermore, the capacity fading in the first few cycles is accompanied by a gradual increase in CE (e.g., rising to 95%/98% at the 5^th^/9^th^ GCD cycles), which is closely associated with the formation and evolution of Zn_x_(OH)_y_Cl_z_ (**Figure S23**). Quantitative XPS analysis of Zn 2p_3/2_ spectra reveal the compositional evolution of the deposited Zn_x_(OH)_y_Cl_z_ (**Figure S23a**): the ratio of OH^−^ to Cl^−^ increases from 1:4 (at the 5^th^ cycle) to approximately 3.6:1 (at the 50^th^ cycle). Additionally, DRT analysis of EIS data identifies a τ_2_ peak at time constant of ~10^-4^ s, which confirms the presence of SEI (Zn_x_(OH)_y_Cl_z_) (**Figure S23b-c**).^32,33^ Notably, the significant increase in impedance and the high-frequency shift of the τ_2_ peak from the 1^st^ to 5^th^ cycles indicate that the thickness of the Zn_x_(OH)_y_Cl_z_ layer increases along with its compositional variation. Collectively, the gradual promotion of CE and the progressive increase of OH^−^ ratio in SEI provide strong evidence that the irreversible formation and dynamic evolution of Zn_x_(OH)_y_Cl_z_ are the primary factors contributing to capacity fading in the initial stage.

For the long-term cycling stage (>20th cycle), the battery maintains a high average CE of 99.5%, while a prominent capacity loss is observed in the high-potential region of the GCD profiles (**Figure S22b** and **S22d**). This phenomenon demonstrates that the capacity decline in this stage is mainly attributed to undesired side reactions between the highly reactive radical cation (PDPZ^x+^) and oxygenic intermediates (e.g., ·OH, ·O_2_^2−^) via free-water oxidation at elevated potentials, which is supported by relevant studies.^20,34^ As a result, the final retained capacity (~100 mAh g^−1^) is mainly derived from the low-potential plateau of the GCD profiles. Consistent with this conclusion, DRT analysis shows no obvious variation in the τ_2_ peak after the 20th cycle, indicating that the Zn_x_(OH)_y_Cl_z_ layer tends to stabilize and the side reactions related to SEI formation gradually diminish. Therefore, the dominant factor for capacity loss during long-term cycling is the parasitic reactions of PDPZ under high potential polarization, rather than the contribution of Zn_x_(OH)_y_Cl_z_ formation.

**Figure S24.** (a) GCD profiles of PDPZ cathode in three electrode system (*vs.* Hg/Hg_2_SO_4_) during activation cycles and (b) evolution of GCD profiles during the long-term cycling in 30 m ZnCl_2_ at the current densities of 0.2 A g^−1^.

**Notes to Figure S24:** The activation of PDPZ electrode in 30 m ZnCl_2_ stems from the high viscosity (∼318.5 mPa·s) and low ionic conductivity (∼12.7 mS cm^−1^) of the concentrated ZnCl_2_ electrolyte,^35,36^ which retard electrolyte penetration into the electrode and slow ion diffusion kinetics.^37,38^ Progressive electrode wetting during cycling enhances active site utilization, leading to the characteristic capacity increase.

**Figure S25.** GCD profiles and rate performance of PDPZ in 10 m ZnCl_2_ at current densities ranging from 0.1 to 1.0 A g^−1^.

**Figure S26.** Electrochemical kinetics. (a) Cyclic voltammetry (CV) curves measured at scan rates ranging from 0.2 to 1 mV s^−1^; (b) Linear correlation between log(v) and log(i) at the redox peak potentials.

**Figure S27.** Galvanostatic intermittent titration technique (GITT) profiles for PDPZ at the current densities of 0.2 A g^−1^.

**Figure S28.** Electrochemical performance of chloride-based dual-ion batteries (Cl-DIBs) with Zn||PDPZ full-cell configuration. (a) Schematic configuration of the fabricated Cl-DIB full cell. The GCD profiles of the initial five cycles at the current densities of 0.1 A g^−1^ in (b) 10 m ZnCl_2_ and (c) 30 m ZnCl_2_. (d) Comparison of the rate capability at current densities ranging from 0.2 to 2.0 A g^−1^ in 10 m ZnCl_2_ and 30 m ZnCl_2_. (e) GCD profiles and rate performance at current densities ranging from 0.2 to 2.0 A g^−1^ in 10 m ZnCl_2_. (f) GCD profiles and rate performance at current densities ranging from 0.2 to 2.0 A g^−1^ in 30 m ZnCl_2_. (g) Comparison of the cycling stability at the current densities of 0.2 A g^−1^ in 10 m ZnCl_2_ and 30 m ZnCl_2_. (h) Cycling stability at 1.0 A g^−1^ with corresponding GCD profiles shown in (i).

**Figure S29.** Electrochemical Cl^−^ storage performance of PDPZ (magenta) compared with reported p-type organic (pink) and inorganic (yellow) cathodes in aqueous ZnCl_2_ electrolyte, including (a) specific capacity, (b) energy density, and (c) calendar life.^39–48^ Performance of representative p-type organic cathodes for polyatomic anion storage in Zn^2+^-based aqueous electrolytes is also included in green for comparison.^49–56^

**Figure S30.** (a) Analysis of the discharge plateau voltage range and discharge plateau capacity of the PDPZ electrode in Zn||PDPZ Cl-DIBs using 30 m ZnCl_2_ electrolyte. (b) Comparison of discharge plateau of PDPZ with recently reported p-type organic cathode in aqueous zinc-ion batteries. (c) Comparison of cutoff voltage of PDPZ with other p-type organic and inorganic/carbon cathodes in Zn^2^⁺ aqueous electrolytes, together with typical p-type organic cathodes for anion storage in Li⁺ nonaqueous electrolytes.^1,25,57–64^

# 6. **Cl^−^ Storage Mechanism of PDPZ**

**Figure S31.** *Ex-situ* X-ray photoelectron spectroscopy (XPS) survey spectra of PDPZ electrode before and after Cl^−^ intercalation.

**Figure S32.** The elemental mapping and corresponding energy-dispersive X-ray spectroscopy (EDS) data of PDPZ electrode acquired in the charged state after undergoing GCD cycling.

**Figure S33.** EQCM and charge carrier analysis during (a) charging and (b) discharging at 3 mV s^−1^. Dots, cyclic profiles of mass change; magenta line, electrode potential versus charge. Δ*m/Q* values derived from linear regression (slope fitting) for individual redox reaction steps during the charging process. Δ*m/Q* quantifies the mass variation per unit charge transfer, reflecting the mass of ions and/or solvents insertion/desertion at each reaction stage.

**Figure S34.** The elemental mapping and corresponding EDS data of PDPZ electrode acquired in the discharged state after undergoing GCD cycling.

**Figure S35.** High-resolution XPS spectra of Zn 2p for PDPZ electrodes at different charge states. Blue, as prepared electrode; grey, charged electrode; pink, discharged electrode.

**Figure S36.** High-resolution (a) Zn 2p_3/2_, (b) O 1s, (c) Cl 2p_3/2_, and (d) Zn 3p XPS spectra of cycled PDPZ electrode (fully discharged, 50^th^ cycle, in 10 m ZnCl_2_)

**Figure S37.** (a) High-resolution Zn L_3_M_4,5_M_4,5_ XPS spectra of cycled PDPZ electrode (fully discharged, 50 cycles, in 10 m ZnCl_2_); (b) Comparison of the Zn L_3_M_4,5_M_4,5_ kinetic energies between Zn_x_(OH)_y_Cl_z_ species on cycled PDPZ electrode surfaces and selected Zn(II) compounds. Values in parentheses denote the sample sizes used for calculating the mean and standard deviations; (c) Wagner plot of Zn 2p_3/2_–Zn L_3_M_4,5_M_4,5_ transitions for comparing the Zn_x_(OH)_y_Cl_z_ species on the surface of cycled PDPZ electrode with selected Zn(II) compounds. The data for selected Zn(II) compounds is extracted from literature.^65,66^

**Note to Figure S36-S37.** To clarify the chemical species formed, we performed a detailed analysis of the Zn core‑level (*2p, 3p*) photoelectron and Zn L_3_M_4,5_M_4,5_ Auger spectra from the cycled PDPZ electrode. The fitting parameters were systematically compared with literature data for relevant reference compounds, including Zn_5_(OH)_8_Cl_2_·H_2_O, Zn_5_(CO_3_)_2_(OH)_6_, Zn(OH)_2_, and ZnCl_2_ (see **Table S6** for a detailed comparison). Peak deconvolution of the high‑resolution Zn 2p_3/2_ spectrum (peak maximum at 1022.2 eV) reveals two components at binding energies (BEs) of 1021.8 and 1022.8 eV (**Figure S36a**), which we assign to Zn–OH and Zn–Cl bonding, respectively. This assignment is supported by the corresponding Cl 2p_3/2_ and O *1s* signals at 198.5 and 532.3 eV (**Figure S36b–c**), matching reference values from the literature (**Table S6**). Deconvolution of the Zn 3p_3/2_ spectrum also shows two components at 89.3 and 90.4 eV (**Figure S36d**), with an area ratio of 3.6:1. This ratio aligns well with that obtained from the Zn–OH and Zn–Cl components in the Zn 2p_3/2_ spectrum, providing self‑consistent evidence for the coexistence and relative abundance of these two Zn bonding environments. The full width at half‑maximum (FWHM) of the two Zn 2p_3/2_ components (1.89 eV for Zn–OH and 1.78 eV for Zn–Cl) is broader than that of pristine Zn(OH)_2_ (1.7 eV) and ZnCl_2_ (1.6 eV),^65^ suggesting a more complex chemical environment.

To quantify the surface composition, we considered mixed Zn(OH)_x_ and ZnCl_y_ contributions. Based on the overall Zn:Cl atomic ratio (1.71) from XPS survey spectra and the Zn–Cl peak area percentage (21.9%) from Zn 2p_3/2_ deconvolution, the Zn:Cl ratio within the chloride‑containing component is calculated as 0.37:1. This value deviates significantly from the 0.5:1 stoichiometry expected for pure ZnCl2. Together, the distinct yet correlated Zn–OH/Zn–Cl components, the broadened FWHM, and the non‑stoichiometric Zn:Cl ratio collectively point to the formation of a zinc hydroxide chloride phase with a mixed Zn–(OH, Cl) coordination, rather than separate Zn(OH)_2_ or ZnCl_2_ phases.

The spectrum was deconvoluted into four characteristic Auger multiplet peaks (^1^*S*, ^1^*G*, ^3^P/^1^*D*, and ^3^*F*), indexed according to quasi‑atomic coupling of the final‑state holes (**Figure S37a**), along with an associated satellite feature between the ^1^*G* and ^1^*S* peaks, consistent with literature reports.^67–69^ The corresponding fitting parameters are summarized in **Table S7**. The dominant peak (^1^*G* multiplet of the d8 final state) appears at a kinetic energy of 986.4 eV (binding energy = 500.2 eV), confirming the presence of Zn(II) species (**Figure S37b**). However, due to spectral overlap among various Zn(II) compounds and the broad range of reported kinetic energies (*e.g.,* 987.2 ± 0.9 eV for ZnCl_2_), definitive speciation based solely on this peak position remains challenging. To resolve this ambiguity, we employed Wagner (chemical state) plot analysis (**Figure S37c**), which correlates photoelectron binding energy with Auger kinetic energy and is effective for discriminating chemical states. The modified Auger parameter (*α*') derived from this plot (**Table S6**) serves as a sensitive indicator of the chemical environment. The *α*' value for the Zn(II) species on our cycled PDPZ electrode is 2008.7 eV. This value differs significantly from those of pure ZnCl_2_ and Zn(OH)_2_. It is also notably lower than the reported value for crystalline Zn_5_(OH)_8_Cl_2_·H_2_O (2009.43 eV), indicating that the stoichiometry of the surface species formed on our electrode differs from that of the known zinc hydroxychloride.

# 7. **Theoretical Calculations and Experimental in-situ ATR-FTIR/** **UV-vis-NIR Spectra**

**Figure S38.** Comparative analysis of the calculated infrared spectra (unscaled) for d-DPZ^2+^ and d-DPZ^4+^ cations and their respective chloride complexes (d-DPZ^2+^·2Cl^−^ and d-DPZ^4+^·4Cl^−^).

**Note to Figure S38:** The calculated vibrational frequency shifts elucidate the influence of Cl^−^‑π^+^ interactions on the vibrational modes of the d‑DPZ skeleton. Vibrational assignments for the CCN/CNC bending modes and aromatic C–H out‑of‑plane bending vibrations were determined on the basis of spectral calculations and analyses presented in  in **Figure S42-S44** and **Table S8.**

**Figure S39.** *In-situ* attenuated total reflectance Fourier-transform infrared (ATR-FTIR) spectral evolution of the PDPZ electrode during GCD cycling at various states of charge (SOC) (a→d and d→h denoted as the 1^st^ and 2^nd^ GCD plateaus, respectively; spectral range, 1600–650 cm^−1^).

**Figure S40.** Structural evolution of the PDPZ electrode monitored during GCD cycling. *In-situ* FTIR spectral overview recorded at different SOC during GCD operation, showing characteristic vibrational bands in two wavenumber spectral regions (3800–2600 cm^−1^ and 1200–650 cm^−1^).

**Figure S41.** Comparative analysis of the experimental (exp.) and calculated (calc.) FTIR spectra of PDPZ. The simulated frequencies were scaled with a scaling factor of 0.964.^70^

**Figure S42.** Calculated infrared spectrum (unscaled) and representative vibrational mode assignments for d-DPZ in the frequencies of 1700–1100 cm^–1^. Calculated eigenvectors for characteristic vibrational modes at 1548, 1522, 1377, and 1319 cm^–1^ are shown in top-down (left) and frontal (right) views.

**Figure S43.** Calculated infrared spectrum (unscaled) and representative vibrational mode assignments for d-DPZ^2+^·2Cl^−^ in the frequencies of 1700–1100 cm^–1^. Calculated eigenvectors for characteristic vibrational modes at 1516, 1390 and 1267 cm^–1^ are shown in top-down (left) and frontal (right) views.

**Figure S44.** Calculated infrared spectrum (unscaled) and representative vibrational mode assignments for d-DPZ^4+^·4Cl^−^ in the frequencies of 1700–1100 cm^–1^. Calculated eigenvectors for characteristic vibrational modes at 1584, 1549, 1460, 1391 and 1300 cm^–1^ are shown in top-down (left) and frontal (right) views.

**Figure S45.** Theoretical UV-vis-NIR absorption spectra of d-DPZ and coordination states with different anions d-DPZ^2+^·2Cl^−^, d-DPZ^4+^·4Cl^−^, d-DPZ^4+^·4OTf^−^, and d-DPZ^4+^·4TFSI^−^. All the spectra were calculated using time-dependent density functional theory (TD-DFT) method of B3LYP/6-311+G(d) level of theory based on optimized structures. The calculations considered the first 30 excited states and employed the polarizable continuum model (PCM) as the solvation model.

**Figure S46.** TD-DFT calculated UV-vis-NIR absorption profiles with representative frontier molecular orbital distributions: (a) d-DPZ^2+^ and (b) d-DPZ^4+^. H and L represent HOMO and LUMO, respectively.

**Figure S47.** TD-DFT calculated UV-vis-NIR absorption profiles with representative frontier molecular orbital distributions: (a) d-DPZ^4+^·4OTf^‒^ and (b) d-DPZ^4+^·4TFSI^‒^. H and L represent HOMO and LUMO, respectively.

**Figure S48.** (a) Differential *in-situ* UV-vis-NIR absorption spectra of the PDPZ electrode at selected discharge states. The data acquisition points synchronized to the potential markers shown in the discharge profile of Figure 4f. (b) Experimental normalized UV-vis absorption spectra recorded in different electrolyte environments (10 m ZnCl_2_, 5 m ZnCl_2_ in EG, and 3 m Zn(OTf)_2_).

**Figure S49.** In-situ UV-vis-NIR absorbance spectra of PDPZ electrode during galvanostatic cycling in (a) 5 m ZnCl_2_ in EG and (b) 3 m Zn(OTf)_2_ electrolytes.

**Note to Figure** **S4****5–S49.** To unambiguously establish the causal link of the observed ICT transitions to the Cl⁻ anion, we performed complementary theoretical calculations ((**Figure** **S45–S47**) and control experiments (**Figure** **S48–S49** and **Table S9**).

TD-DFT calculations supplement UV-vis-NIR absorption analyses for cationic species (d-DPZ^2+^ and d-DPZ^4+^) and polyatomic-anion-coordinated complexes (d-DPZ^4+^·4OTf^−^/4TFSI^−^) (**Figure R1-8a, b**). For isolated d-DPZ^2+^ and d-DPZ^4+^ lacking anions, ICT from anions is precluded. Although exhibiting absorption bands at 510–900 nm (**Figure S46**), their primary electronic transitions involve: (i) bridging benzene→pyrazine ring transition (d-DPZ^2+^: HOMO−2→LUMO, *λ* = 671.27 nm, *f* = 0.0562), and (ii) intrapyrazine ring transitions (d-DPZ^4+^: HOMO−5→LUMO and HOMO−4→LUMO+1, *λ* = 613.36 nm, *f* = 0.0986). Upon Cl⁻ coordination forming d-DPZ^4+^·4Cl^−^, due to the significant Cl⁻→π⁺ ICT between the d-DPZ^4+^ skeleton and Cl⁻ (*λ* = 850.08 nm), the absorption band of d-DPZ^4+^·4Cl^−^ significantly red-shifted as compared with d-DPZ^4+^, which is in good agreement with the experimental in-situ UV-vis-NIR spectrum of PDPZ in the fully charged state in 10 m ZnCl_2_ aqueous electrolyte. Compared to d-DPZ^4+^·4Cl^−^, complexes with polyatomic anions (d-DPZ^4+^·4OTf^−^ and d-DPZ^4+^·4TFSI^−^) exhibit blue-shifted absorption bands (**Figure R1-8b**). For d-DPZ^4+^·4OTf^−^, the lowest-energy absorption is primarily contributed by ICT from OTf^−^ anion to d-DPZ^4+^ skeleton (*λ* = 551.01 nm, 616.24 nm), whereas in d-DPZ^4+^·4TFSI^−^ it stems from the electron transition within the aromatic skeleton (*λ* = 624.29 nm). These spectral features and energies differ markedly from the Cl⁻→π⁺ ICT observed in d-DPZ^4+^·4Cl^−^.

To probe the specificity of the interaction between the oxidative PDPZ and chloride ions, *in situ* electrochemical UV-vis-NIR spectra were further performed in 3 m Zn(OTf)_2_ aqueous electrolyte and non-aqueous 5 m ZnCl_2_ (in ethylene glycol (EG)) (**Figure S48–S49**). In Zn(OTf)_2_ (aq), the near-infrared absorption band of fully charged PDPZ exhibits a ~15 nm blue-shift versus the 10 m ZnCl_2_(aq) reference, aligning with theoretical predictions of ICT blue-shift. The smaller experimental shift magnitude (vs. computational data) may ascribe to the electrolyte concentration effects. Experimental investigation in 10 m Zn(OTf)_2_ was precluded by the aqueous solubility limit of Zn(OTf)_2_. In non-aqueous media, chloride-incorporated PDPZ shows visible-to-NIR absorption profiles closely matching those in aqueous ZnCl_2_, eliminating concerns regarding OH^−^ co-intercalation interference in spectral assignments.

Therefore, comparative TD-DFT and experimental analysis confirms that the near-infrared absorption in *in situ* UV-vis-NIR spectra of PDPZ electrode in aqueous ZnCl_2_ linked explicitly to Cl⁻→π⁺ ICT within PDPZ^x+^·xCl^−^.

# 8. **Supporting Tables**

**Table S1.** The calculated Gibbs free energy changes for per electron (*ΔG/e*) of PAAs and corresponding ion pairs.

| Compounds | Electronic Energy  (hartree) | Thermal Correction  (hartree) | Free Energy (hartree) | Reaction processes | *ΔG*/e (eV) |
| --- | --- | --- | --- | --- | --- |
| d-TPA | −1498.603 | 0.470 | −1498.133 | / | / |
| d-DPZ | −1838.036 | 0.532 | −1837.504 | / | / |
| Cl^−^ | −460.415 | −0.015 | −460.430 | / | / |
| TFSI^−^ | −1827.699 | −0.008 | −1827.691 | / | / |
| OTf^−^ | −961.821 | −0.006 | −961.827 | / | / |
| d-TPA^2+^·2Cl^−^ | −2419.065 | 0.466 | −2418.599 | d-TPA+2Cl^−^ → d-TPA^2+^·2Cl^−^ | 5.36 |
| d-TPA^2+^·2TFSI^−^ | −5153.665 | 0.531 | −5153.134 | d-TPA+2TFSI^−^ → d-TPA^2+^·2TFSI^−^ | 5.18 |
| d-TPA^2+^·2OTf^−^ | −3421.896 | 0.501 | −3421.395 | d-TPA+2OTf^−^ → d-TPA^2+^·2OTf^−^ | 5.33 |
| d-DPZ^2+^·2Cl^−^ | −2758.530 | 0.533 | −2757.997 | d-DPZ+2Cl^−^ → d-DPZ^2+^·2Cl^−^ | 4.99 |
| d-DPZ^4+^·4Cl^−^ | −3679.010 | 0.526 | −3678.484 | d-DPZ^2+^·2Cl^−^+2Cl^−^ → d-DPZ^4+^·4Cl^−^ | 5.07 |
|  |  |  |  | d-DPZ+4Cl^−^ → d-DPZ^4+^·4Cl^−^ | 5.03 |
| d-DPZ^4+^·4TFSI^−^ | −9148.217 | 0.665 | −9147.552 | d-DPZ+4TFSI^−^ → d-DPZ^4+^·4TFSI^−^ | 4.87 |
| d-DPZ^4+^·4OTf ^−^ | −5684.676 | 0.597 | −5684.079 | d-DPZ+4OTf ^−^ → d-DPZ^4+^·4OTf ^−^ | 4.99 |

**Notes to Table S1:** To compare the thermodynamic coordination behaviors of different anions, we evaluated the free energy per electron (ΔG/e) between PAA^x+^ and Cl^−^, OTf^−^ and TFSI^−^. All PAA^x+^·xOTf^−^/xTFSI^−^ complexes exhibited less positive free energies than their corresponding PAA^x+^·xCl^−^ counterparts, demonstrating the weakly nucleophilic polyatomic anions (OTf⁻ and TFSI⁻) bind more readily to PAA^x+^ via Coulombic attraction from a thermodynamic perspective.

**Table S2** Values of Electron Density (ρ(*r*)) and its Laplacian (∇^2^ρ(*r*)) at the BCPs of d-TPA^2+^ and d-DPZ^x+^ with Cl^−^ in complex. ^[a]^

| Complex | ρ(r) | ∇^2^ρ(r) |
| --- | --- | --- |
| d-TPA^2+^·2Cl^−^ | 0.00637 (C-Cl), 0.00637 (C-Cl),  0.01082 (H-Cl), 0.01082 (H-Cl)  **0.03438** | 0.01772 (C-Cl), 0.01772 (C-Cl),  0.03366 (H-Cl), 0.03366 (H-Cl)  **0.10276** |
| d-DPZ^2+^·2Cl^−^ | 0.00596 (C-Cl), 0.00596 (C-Cl),  0.00800 (H-Cl), 0.00576 (N-Cl),  0.00598 (N-Cl), 0.00683 (H-Cl),  0.00683 (H-Cl), 0.00598 (N-Cl),  0.00576 (N-Cl), 0.00800 (H-Cl),  0.00596 (C-Cl), 0.00596 (C-Cl)  **0.07698** | 0.01812 (C-Cl), 0.01812 (C-Cl),  0.02360 (H-Cl), 0.01791 (N-Cl)  0.01851 (N-Cl), 0.01962 (H-Cl),  0.01962 (H-Cl), 0.01851 (N-Cl),  0.01791 (N-Cl), 0.02360 (H-Cl),  0.01812 (C-Cl), 0.01812 (C-Cl)  **0.23176** |
| d-DPZ^2+^·4Cl^−^ | 0.01264 (C-Cl), 0.00466 (H-Cl),  0.00554 (H-Cl), 0.01288 (C-Cl),  0.00375 (H-Cl), 0.00563 (H-Cl),  0.00563 (H-Cl), 0.00375 (H-Cl),  0.01288 (C-Cl), 0.00554(H-Cl),  0.00466 (H-Cl), 0.01264 (C-Cl)  **0.09020** | 0.03843 (C-Cl), 0.01284 (H-Cl),  0.01563 (H-Cl), 0.03884 (C-Cl),  0.01020 (H-Cl), 0.01590 (H-Cl),  0.01590 (H-Cl), 0.01020 (H-Cl),  0.03884 (C-Cl), 0.01563 (H-Cl),  0.01284 (H-Cl), 0.03843 (C-Cl)  **0.26368** |

[a] Sum of ρ(*r*) and ∇^2^ρ(*r*) is given in bold.

**Table S3** Energy decomposition analysis (EDA) of d-PAA^x+^ with Cl^−^, TFSI^−^, and OTf^−^ by using the method of sobEDA.^[a]^ (in kcal/mol)

| Complex | Δ*E*_int_ | Δ*E*_xrep_ | Δ*E*_att_^[b]^ | Δ*E*_els_ | Δ*E*_orb_ | Δ*E*_DFTc_ | Δ*E*_dc_ | Δ*E*_int/A⁻_ | Δ*E*_xrep/A⁻_ | Δ*E*_att/A⁻_ | Δ*E*_els/A⁻_ | Δ*E*_orb/A⁻_ | *ΔE*_els/A⁻_ /*ΔE*_att/A⁻_ | *ΔE*_orb/A⁻_ /*ΔE*_att/A⁻_ | % ionic character^[c]^ | % covalent character^[c]^ |
| --- | --- | --- | --- | --- | --- | --- | --- | --- | --- | --- | --- | --- | --- | --- | --- | --- |
| d-TPA^2+^·2Cl^−^ | -239.8 | 30.6 | -300.4 | -215.0 | -40.3 | -8.0 | -7.1 | -119.9 | 15.3 | -150.2 | -107.5 | -20.1 | 71.6% | 13.4% | 84.2% | 15.8% |
| d-TPA^2+^·2TFSI^−^ | -219.0 | 47.8 | -266.8 | -197.0 | -26.5 | -17.0 | -26.2 | -109.5 | 23.9 | -133.4 | -98.5 | -13.2 | 73.8% | 9.9% | 88.2% | 11.8% |
| d-TPA^2+^·2OTf^−^ | -219.9 | 39.8 | -259.7 | -202.6 | -26.4 | -13.6 | -17.1 | -109.9 | 19.9 | -129.8 | -101.3 | -13.2 | 78.0% | 10.2% | 84.4% | 15.6% |
| d-DPZ^2+^·2Cl^−^ | -241.6 | 41.8 | -283.4 | -228.5 | -33.6 | -11.1 | -10.1 | -120.8 | 20.9 | -141.7 | -114.2 | -16.8 | 80.6% | 11.8% | 87.2% | 12.8% |
| d-DPZ^4+^·4Cl^−^ | -932.9 | 110.2 | -1043.1 | -891.2 | -104.6 | -24.7 | -22.7 | -233.2 | 55.1 | -260.8 | -222.8 | -26.1 | 85.4% | 10.0% | 89.5% | 10.5% |
| d-DPZ^4+^·4TFSI^−^ | -873.2 | 126.6 | -999.8 | -793.2 | -95.5 | -42.4 | -68.7 | -218.3 | 63.3 | -249.9 | -198.3 | -23.9 | 79.3% | 9.5% | 89.3% | 10.7% |
| d-DPZ^4+^·4OTf^−^ | -881.2 | 107.7 | -988.9 | -822.3 | -87.6 | -34.0 | -45.0 | -220.3 | 53.8 | -247.2 | -205.6 | -21.9 | 83.1% | 8.8% | 90.4% | 9.6% |

[a] Δ*E*_int_, Δ*E*_els_, Δ*E*_xrep_, Δ*E*_orb_, Δ*E_DFT_*_c_ and Δ*E_d_*_c_ denote the total interaction energy, electrostatic energy, exchange-repulsion energy, orbital interaction energy, coulombic correlation energy, DFT correlation energy, and dispersion correction energy, respectively. Δ*E*_/A⁻_ denote the average interaction energy per anion (Cl^−^, TFSI^−^, and OTf^−^).^71^

[b] Δ*E*_att_ is the total attraction energy, Δ*E*_att_=Δ*E*_els_+Δ*E*_orb_+Δ*E_DFT_*_c_ +Δ*E_d_*_c_.

[c] The percentages of covalent and ionic character contributions can be quantitatively assessed using the following ratios derived from the EDA components:^72^

$$\text{\% covalent}\text{ }\text{=}\text{ }\frac{{\text{∆}\text{E}}_{\text{orb}}}{{\text{∆}\text{E}}_{\text{orb}}\text{+}{\text{∆}\text{E}}_{\text{els}}}\text{ Equation (1) }$$

$$\text{\% ionic}\text{ }\text{=}\text{ }\frac{{\text{∆}\text{E}}_{\text{elstat}}}{{\text{∆}\text{E}}_{\text{orb}}\text{+}{\text{∆}\text{E}}_{\text{els}}}\text{ Equation (2) }$$

**Notes to Table S3:** From the energy decomposition analysis (EDA) results, PAA^x+^·xCl^−^ complexes exhibit substantially larger Δ*E*_int_ arising from ionic-covalent interactions compared to the noncovalent interactions observed in PAA^x+^·xOTf⁻ and PAA^x+^·xTFSI⁻ complexes with the same electron acceptors. Specifically, in PAA^x+^·xCl^−^ complexes, the value of Δ*E*_dc_/Δ*E*_att_ (2.2%~3.6%) is substantially lower than that of Δ*E*_orb_/Δ*E*_att_ (10.0%~13.4%). However, compare to polyatomic anions-based complexes, larger Δ*E*_dc_/Δ*E*_att_ (4.8%~9.8%) and lower Δ*E*_orb_/Δ*E*_att_ (8.8%~10.2%) are observed in PAA^x^⁺·xOTf⁻ and PAA^x^⁺·xTFSI⁻. This result confirmed the distinction between the single-point and multicenter interactions between π-acceptors and Cl^−^ vs. OTf⁻/TFSI⁻, respectively.

**Table S4.** Atomic dipole corrected Hirshfeld (ADCH) atomic charge analysis of d-TPA^2+^·2Cl^−^, d-DPZ^2+^·2Cl^−^, and d-DPZ^4+^·4Cl^−^.

| Complex | Isolated PAA^x+^ ion^[a]^ | Fragment ADCH charge  (PAA^x+^ in PAA^x+^Cl^−^ complex) | ΔADCH charge^[b]^  PAA^x+^→ PAA^x+^·xCl^−^ |
| --- | --- | --- | --- |
| d-TPA^2+^·2Cl^−^ | 2.000 | 1.192 | −0.808 |
| d-DPZ^2+^·2Cl^−^ | 2.000 | 1.264 | −0.736 |
| d-DPZ^4+^·4Cl^−^ | 4.000 | 2.408 | −1.592 |

[a] The notation "PAA^x+^" is assigned to (d-TPA^2+^, d-DPZ^2+^, and d-DPZ^4+^).

[b] The ΔADCH charge was calculated based on the change in atomic charge from the isolated X^+^ ion to the fragment PAA^x+^ in PAA^x+^·xCl^−^ complex.

**Table S5.** Comparison of electrochemical Cl⁻ storage performance of PDPZ with reported p-type organic and inorganic cathodes in aqueous ZnCl_2_ electrolyte, together with typical p-type organic cathodes for polyatomic anion storage in Zn^2^⁺ aqueous electrolytes.

|  | **Cathode materials** | **Electrolyte** | **Charge carrier** | **Cutoff voltage (V)** | **Specific capacity**  **(mAh g^−1^)** | **Energy density @ Power density**  **(Wh kg^−1^ @ kW kg**^−^**^1^)** | **Calendar life^a^** | **Ref** |
| --- | --- | --- | --- | --- | --- | --- | --- | --- |
| Poly(arylamine)s |   PDPZ | 10 m ZnCl_2_ | Cl^−^ | −1.1−0.65^b^ | 181 @ 0.1 A g^−1^  99 @ 1 A g^−1^ | / | 54%, 870 h, 0.2 A g^−1^ | This work |
|  |  |  | Cl^−^ | 0−1.9 | 194 @ 0.1 A g^−1^  80 @ 2 A g^−1^ | 163 @ 0.08 | 60%, 182 h, 0.2 A g^−1^ |  |
|  |  | 30 m ZnCl_2_ | Cl^−^ | −1.1−0.65^b^ | 155 @ 0.2 A g^−1^ | / | 85%, 775 h, 0.2 A g^−1^ |  |
|  |  |  | Cl^−^ | 0−1.9 | 236 @ 0.1 A g^−1^  105 @ 2 A g^−1^ | 175 @ 0.07 | 55%, 198 h, 0.2 A g^−1^ |  |
|  |   m-PTPA/Conjugated micoporous Polymer (CMP) | 2 M ZnCl_2_ | Cl^−^ | 0.6–1.7 | 210.7 @ 0.5 A g^−1^  107.5 @ 6 A g^−1^ | 236 @ 0.6  122.5 @ 6.8 | 87.6%, 36 h, 6 A g^−1^ | *39* |
| CMP |   CLPy | 30 m ZnCl_2_ | Cl^−^ | 0.6−1.8 | 180 @ 0.05 A g^−1^  105 @ 3A g^−1^ | 145 @ 0.06^a^ | 96.4%, 2600 h, 3 A g^−1^ | *40* |
| Polyindole |   Polyindole | 1 m ZnCl_2_ | Cl^−^ | 1.0−2.0 | 81 @ 500 A m^−2^  60 @ 1000 A m^−2^ | 89 | / | *41* |
| Nitronyl nitroxide derivatives |   PTVE | 0.1 M ZnCl_2_ + 0.1 M NH_4_Cl | Cl^−^ | 1.4−2.0 | 131 @ 60 C | 222.7 @ 60 C^a^ | / | *42* |
| Prussian blue | PBAR^c^ | 10 m ZnCl_2_ | [ZnCl_4_]^2−^ | 0.2−1.4^a^ | 66 @ 0.066 A g^−1^  56 @ 0.264 A g^−1^ | 23 | 26%, 42 h, 0.264 A g^−1^ | *43* |
|  | Zn_3_[Fe(CN)_6_]_2_ | 30 m ZnCl_2_ | [ZnCl_4_]^2−^ | 0−1.6^a^ | ∼30 @ 0.03 A g^–1^ | ∼28.5 | 58%, 94 h, 0.53 A g^−1^ | *44* |
| Metal-oxide | WO_3_ | 30 m ZnCl_2_ | Zn^2+^ | 0–1.2 | 94.8 @ 0.1 A g^−1^ | 18.9 @ 0.75 | 94.8%, 132 h, 1.0 A g^−1^ | *45* |
|  | Mn_3_O_4_ | 30 m ZnCl_2_ | [ZnCl_4_]^2−^ | 1.2−1.9 | 200 @ 0.05 A g^−1^ | 150 | 99%, 400 h, 0.05 A g^−1^ | *46* |
| Carbon | Graphite^b^ | 30 m ZnCl_2_ + 120 *m* ChCl + 5 *m* KI | I-Cl | -0.2−1.4  (vs. SHE) | 291 @ 0.03 A g^–1^ | 108 | 89%, 776 h, 0.03 A g^−1^ | *47* |
|  | N-doped graphene^b^ | 30 m ZnCl_2_ | [ZnCl_x_]^2−x^ | 0.8−1.95 | 134 @ 0.1 A g^−1^ | 180.9 @ 0.1^a^ | 61%, 2144 h, 0.1 A g^−1^ | *48* |
| CMP |   PHOs | 3 M ZnSO_4_ | SO_4_^2−^ | 0.3−1.7^a^ | 276 @ 0.1 A g^−1^  111 @ 50 A g^−1^ | 317 | 71.4%, 3199 h, 10 A g^−1^ | *49* |
|  |  | 3 M Zn(OTf)_2_ | OTf^−^ |  | 233 @ 0.1 A g^−1a^  54 @ 50 A g^−1a^ | 273 | 67.1%, 2181 h, 10 A g^−1^ |  |
|  |  | 3 M Zn(TFSI)_2_ | TFSI^−^ |  | 173 @ 0.1 A g^−1a^  16 @ 50 A g^−1a^ | 210 | 60.2%, 1257 h, 10 A g^−1^ |  |
| Arylamine derivatives |   BBOPO | 3 M Zn(OTf)_2_ | OTf^−^ | 0.1-2.0 | 210 @ 0.5 A g^−1^  105 @ 20 A g^−1^ | / | 80.3%, 125 h, 3 A g^−1^ | *50* |
|  |   PSe | 3 M Zn(OTf)_2_ | OTf^−^ | 0–1.8 | 227 @ 0.1 A g^−1^  141 @ 10 A g^−1^ | 273 | 86%, 1640 h, 2 A g^−1^ | *51* |
|  |   DHTAT | 3 M Zn(ClO_4_)_2_ | ClO_4_^−^ | 0.2−1.8 | 200.3 @ 0.1 A g^−1^  88.5 @ 10 A g^−1^ | 105.1 | 73%, 183 h, 5 A g^−1^ | *52* |
|  |   dNPC | 20 m LiTFSI +  1 m Zn(TFSI)_2_ | TFSI^−^ | 0.6−1.8 | 100 @ 0.05 A g^−1^  76 @ 0.5 A g^−1^ | 100 @ 0.65 | 96%, 304 h, 0.5 A g^−1^ | *53* |
|  |   BDB | 1 m Zn(OTf)_2_ +  19 m LiTFSl | TFSI^−^ | 0.4−2.1 | 125 @ 0.026 A g^−1^  80 @ 0.078 A g^−1^ | 155 | 82%, 287 h, 0.39 A g^−1^ | *54* |
| Nitronyl nitroxide derivatives |   PTVE | 1 M Zn(SO_4_)_2_ | SO_4_^2−^ | 1.3−1.95^a^ | 77 @ 0.2 A g^−1a^  58 @ 10 A g^−1^ | / | 21.9%, 141 h, 1 A g^−1^ | *55* |
|  |  | 1 M Zn(OTf)_2_ | OTf^−^ |  | 84 @ 0.2 A g^−1a^  52 @ 10 A g^−1^ |  | 77%, 164 h, 1 A g^−1^ |  |
|  |  | 1 M Zn(ClO_4_)_2_ | ClO_4_^−^ |  | 81 @ 0.2 A g^−1a^  50 @ 10 A g^−1^ |  | 91%, 172 h, 1 A g^−1^ |  |
| Triphenylphosphine derivatives |   TP-Se | 1 M Zn(OTf)_2_  Acetonitrile/water, v:v = 85:15 | OTf^−^ | 0.5−2.1^a^ | 72.9 @ 0.5 A g^−1^ | 142 | 85.3%, 1079 h, 2 A g^−1^ | *56* |

[a] The ref does not give the specific value, which is inferred according to the data.

[b] The electrochemical performance was collected in a three-electrode system.

[c] Rhombohedral phase of Zn_3_[Fe(CN)_6_]_2_-based Prussian blue analogues (PBAs).

**Table S6.** Comparison of experimental binding and kinetic energies for selected photoelectrons and Auger electrons with literature values for Zn_5_(OH)_8_Cl_2_·H_2_O, Zn(OH)_2_, and ZnCl_2_.

| **Compound** | **Kinetic Energy (eV)** | Modified Auger parameter (*α*′) | **Binding Energy (eV)** | | | | | | **Zn:Cl**  **atomic ratio** | **Ref.** |
| --- | --- | --- | --- | --- | --- | --- | --- | --- | --- | --- |
|  | Zn L_3_M_4,5_M_4,5_ |  | Zn 2p_3/2_ | | Zn 3p_3/2_ | | Cl 2p_3/2_ | O 1s |  |  |
|  |  |  | Zn-OH | Zn-Cl | Zn-OH | Zn-Cl |  |  |  |  |
| Zn_x_(OH)_y_Cl_z_ | 986.4 | 2008.6 | 1022.2^[a]^ (2.19)^[b^**^]^** | | 89.6^[a]^ | | 198.5  (1.73) | 532.3  (2.2) | 1.7 | This work |
|  |  |  | 1022.0 (1.89) | 1023.0 (1.78) | 89.3  (2.7) | 90.4  (2.0) |  |  |  |  |
| Zn_5_(OH)_8_Cl_2_·H_2_O | 987.2 | 2009.43 | 1022.21^[a]^ | | 89.1^[a]^ | | 198.7 | 531.9 | 2.5 | 66 |
|  |  |  | 1022.1 | ~1023.1 |  |  |  |  |  |  |
|  | / | / | 1023.7^[a]^ | | / | | 198.9 | 531.6 |  | *73* |
|  |  | / | 1022.0 | 1024.1 |  |  |  |  |  |  |
|  | / | / | 1023.1 | | / | | 198.7 | 531.8 |  | *74* |
| Zn_5_(CO_2_)_2_(OH)_6_ | 987.5±0.6 ^[c]^ | / | 1022.2±0.4^[c]^ | | / | | / | / | / | 65 |
|  | 987.6±0.1 | 2009.6 | 1022.0±0.1 (1.9±0.1) | | / | | / | 531.6 |  |  |
| Zn(OH)_2_ | / | / | 1024.6 | | / | | / | 533.5 | */* | *74* |
|  | 987.5 | 2009.31 | 1021.8 | | 88.5^[a]^ | | / | 531.4 |  | *67* |
|  | 987.4 | / | / | | / | | / | 531.6 |  | *75* |
|  | 987.2±0.4^[c]^ | / | 1022.2±0.5^[c]^ | | / | | / | / |  | *65* |
|  | 987.4 | 2009.0 | 1021.6 (1.7) | | / | | / | 531.4 |  |  |
|  | / | / | 1024.2 | | 89.3^[a]^ | | / | 531.1 |  | 76 |
| ZnCl_2_ | / | / | 1023.5 | | / | | 198.9 | / | 0.5 |  |
|  | 987.2±1.9^[c]^ | / | 1022.8±0.8^[c]^ | | / | | / | / |  | *65* |
|  | 986.3±0.2 | 2009.9 | 1023.6±0.3 (1.6) | | / | | 199.7 | / |  |  |

[a] The BE obtained without peak fitting.

[b] FWHM values are provided in parentheses.

[c] The average BE and standard deviation surveyed by Henderson et al., from published literature.

**Table S7.** Peak parameters used for the deconvolution of Zn L_3_M_4,5_M_4,5_ spectra.

| **Peak #** | **BE** | **KE** | **ΔE_BE_ (relative to peak 1G)** | **Attribution** | **Area** | **Area ratio within component** | **FWHM** | **%GL** | **Σχ^2^** |
| --- | --- | --- | --- | --- | --- | --- | --- | --- | --- |
|  | **eV** | **eV** | **eV** |  |  | **%** | **eV** | **%** | **%** |
| 1 | 505.1 | 981.5 | 4.9 | ^1^S | 4000 | 2.7 | 2.5 | 0 | 2.81 |
| 2 | 502.1 | 984.5 | 1.9 | Satellite structure | 12398 | 8.5 | 2.1 | 18 |  |
| 3 | 500.2 | 986.4 | / | ^1^G | 64960 | 44.5 | 2.2 | 0 |  |
| 4 | 498.4 | 988.2 | -1.7 | ^3^P/^1^D | 24737 | 16.9 | 2.5 | 0 |  |
| 5 | 496.7 | 989.9 | -3.5 | ^3^F | 39947 | 27.4 | 3.0 | 30 |  |

**Table S8.** Calculated vibrational wavenumber (cm^−1^, unscaled and scaled), experimental infrared peaks/bands (cm^−1^) and assignments for d-TPA^2+^·2Cl^−^, d-DPZ^2+^·2Cl^−^, and d-DPZ^4+^·4Cl^−^.

| **System** | **ν_calc_^[a]^** | **ν_scal_^[b]^** | **ν_exp_^[c]^** | **ν_exp, power_^[d]^** | **Interpretation** |
| --- | --- | --- | --- | --- | --- |
| PDPZ | 1548 | 1492 | 1503 | 1505 | ν/β/ρ (phenyl, A^R^) + ν (CN, A^R^) |
|  | 1522 | 1467 | 1472 | 1484 | ν/β/ρ (phenyl, A^P^) + β (CCN/CNC, A^P^) |
|  | 1377 | 1327 | 1322 | 1338 | ν/β/ρ (phenyl, A^P^) + β (CCN/CNC, A^P^) |
|  | 1319 | 1271 | 1257 | 1284 | ν/β/ρ (phenyl, A^P^) + β (CCN/CNC, A^P^) |
| d-DPZ^2+^·2Cl^−^  /1^st^ redox step | 1603 | 1545 | 1555-1485 | / | ν/β/ρ (phenyl, A^P^) |
|  | 1516 | 1461 | 1460 | / | ν/β/ρ (phenyl, A^P^) + β (CCN/CNC, A^P^) |
|  | 1390 | 1340 | 1353 | / | ν/β/ρ (phenyl, A^P^) + β (CCN/CNC, A^P^) + ν (CN, A^R^) |
|  | 1267 | 1221 | 1273 | / | ν/β/ρ (phenyl, A^P^) + β (CCN/CNC, A^P^) + ν (CN, A^R^) |
| d-DPZ^4+^·4Cl^−^  /2^nd^ redox step | 1584 | 1527 | 1541-1485 | / | ν/β/ρ (phenyl, A^P^) + β (CCN/CNC, A^P^) |
|  | 1549 | 1493 |  | / | ν/β/ρ (phenyl, A^P^) + β (CCN/CNC, A^P^) + ν (CN, A^P^) |
|  | 1460 | 1407 | 1431 | / | ν/β/ρ (phenyl, A^P^) + β (CCN/CNC, A^P^) |
|  | 1391 | 1341 | 1362 | / | ν/β/ρ (phenyl, A^P^) + β (CCN/CNC, A^P^) + ν (CN, A^P^) |
|  | 1300 | 1253 | 1293 | / | ν/β/ρ (phenyl, A^P^) + β (CCN/CNC, A^P^) + ν (CN, A^P^) |

[a] ν_calc_ is the calculated unscaled frequencies.

[b] ν_scal_ is the calculated frequencies scaled with a scaling factor of 0.964^31^.

[c] ν_exp_ is the infrared peaks/bands extracted from *in-situ* ATR-FTIR spectra.

[d] ν_exp, power_ is the infrared peaks/bands of PDPZ powder samples

[e] Greek letters ν, β, and ρ denote stretching, bending, and swinging vibrations, respectively. A^P^ and A^R^ represent aromatic rings of phenazine unit and linked phenyl group, respectively.

**Table S9.** Computational UV-Vis-NIR absorption spectra of d-DPZ in various charge and coordination states with different anions at the TD-B3LYP/6-311+G (d) level of theory in water. (H: HOMO; L: LUMO)

| **System** | **λ_TD-DFT_ (nm)** | **Oscillator strength**  **(f)** | **Main transition contribution**  **(%)** | **Excitation**  **energy**  ***E* (eV)** |
| --- | --- | --- | --- | --- |
| d-DPZ | 402.25 | 0.0499 | H → L+2 (41.2%)  H−1 → L+3 (40.8%) | 3.0823 |
|  | 327.51 | 0.2162 | H → L+8 (36.5%)  H−1 → L+7 (28.4%) | 3.7857 |
|  | 317.33 | 0.1140 | H−1 → L+7 (31.7%)  H−1 → L+11 (25.2%)  H → L+8 (23.5%) | 3.9071 |
| d-DPZ^2+^ | 671.27 (band 2) | 0.0562 | H−2 → L (99.6%) | 1.8470 |
| d-DPZ^2+^·2Cl^−^ | 850.08 (band 2,  ICT transition) | 0.0064 | H−1 → L (69.8%)  H−3→ L (30.1%) | 1.4585 |
|  | 652.58 (band 2) | 0.0542 | H−8 → L (99.5%) | 1.8999 |
|  | 501.43 (band 1) | 0.0113 | H−13 → L (73.3%)  H−9 → L (12.1%) | 2.4726 |
|  | 429.37 (band 1) | 0.1446 | H−15 → L (57.1%)  H−18 → L (29.8%) | 2.8876 |
|  | 419.70 (band 1) | 0.1353 | H−18 → L (66.7%)  H−15 → L (20.9%) | 2.9541 |
|  | 399.11 (band 1) | 0.0912 | H → L+4 (91.5%) | 3.1065 |
|  | 341.99 (band 1) | 0.0695 | H → L+8 (94.2%) | 3.6254 |
| d-DPZ^4+^ | 613.36 (band 3) | 0.0986 | H−5→ L (51.9%)  H−4 → L+1 (47.6%) | 2.0214 |
| d-DPZ^4+^·4Cl^−^ | 934.67 (band 3) | 0.0007 | H−3→ L (47.5%)  H−2 → L+1 (41.6%) | 1.3265 |
|  | 800.98 (band 3) | 0.0259 | H−1 → L (51.8%)  H → L+1 (41.1%) | 1.5479 |
|  | 723.15 (band 3,  ICT transition) | 0.1775 | H−11 → L (43.9%)  H−10 → L+1 (37.6%) | 1.7145 |
|  | 548.55 (band 3) | 0.1008 | H−13 → L (51.7%)  H−12 → L+1 (47.6%) | 2.2602 |
| d-DPZ^4+^·4OTf^−^ | 616.26 | 0.0431 | H−1 → L (50.4%)  H → L+1 (46.8%) | 2.0120 |
|  | 551.02 | 0.0594 | H−6 → L (48.4%);  H−7 → L+1 (43.3%) | 2.2501 |
| d-DPZ^4+^·4TFSI^−^ | 624.29 | 0.0778 | H → L (64.9%)  H−1 → L+1 (20.6%) | 1.9860 |
|  | 613.84 | 0.0065 | H−1 → L (35.2%)  H → L+1 (28.6%) | 2.0198 |

# 9. **References**

[1]	F. A. Obrezkov, A. F. Shestakov, S. G. Vasil’ev, K. J. Stevenson, P. A. Troshin, *J. Mater. Chem. A* **2021**, *9*, 2864.

[2]	Y. Xiu, A. Mauri, S. Dinda, Y. Pramudya, Z. Ding, T. Diemant, A. Sarkar, L. Wang, Z. Li, W. Wenzel, M. Fichtner, Z. Zhao-Karger, *Angew. Chem. Int. Ed.* **2023**, *62*, e202212339.

[3]	G. Dai, X. Wang, Y. Qian, Z. Niu, X. Zhu, J. Ye, Y. Zhao, X. Zhang, *Energy Storage Mater.* **2019**, *16*, 236.

[4]	M. J. Frisch, G. W. Trucks, H. B. Schlegel, G. E. Scuseria, M. A. Robb, J. R. Cheeseman, G. Scalmani, V. Barone, G. A. Petersson, H. Nakatsuji, X. Li, M. Caricato, A. V. Marenich, J. Bloino, B. G. Janesko, R. Gomperts, B. Mennucci, H. P. Hratchian, J. V. Ortiz, A. F. Izmaylov, J. L. Sonnenberg, Williams, F. Ding, F. Lipparini, F. Egidi, J. Goings, B. Peng, A. Petrone, T. Henderson, D. Ranasinghe, V. G. Zakrzewski, J. Gao, N. Rega, G. Zheng, W. Liang, M. Hada, M. Ehara, K. Toyota, R. Fukuda, J. Hasegawa, M. Ishida, T. Nakajima, Y. Honda, O. Kitao, H. Nakai, T. Vreven, K. Throssell, J. A. Montgomery Jr., J. E. Peralta, F. Ogliaro, M. J. Bearpark, J. J. Heyd, E. N. Brothers, K. N. Kudin, V. N. Staroverov, T. A. Keith, R. Kobayashi, J. Normand, K. Raghavachari, A. P. Rendell, J. C. Burant, S. S. Iyengar, J. Tomasi, M. Cossi, J. M. Millam, M. Klene, C. Adamo, R. Cammi, J. W. Ochterski, R. L. Martin, K. Morokuma, O. Farkas, J. B. Foresman, D. J. Fox, *Gaussian 16 rev. C.01*, Wallingford, CT **2016**.

[5]	P. J. Stephens, F. J. Devlin, C. F. Chabalowski, M. J. Frisch, *J. Phys. Chem.* **1994**, *98*, 11623.

[6]	P. C. Hariharan, J. A. Pople, *Theor. Chim. Acta* **1973**, *28*, 213.

[7]	M. J. Frisch, J. A. Pople, J. S. Binkley, *J. Chem. Phys.* **1984**, *80*, 3265.

[8]	W. J. Hehre, R. Ditchfield, J. A. Pople, *J. Chem. Phys.* **1972**, *56*, 2257.

[9]	S. Grimme, J. Antony, S. Ehrlich, H. Krieg, *J. Chem. Phys.* **2010**, *132*, 154104.

[10]	S. Grimme, S. Ehrlich, L. Goerigk, *J. Comput. Chem.* **2011**, *32*, 1456.

[11]	F. Meng, Y. Bu, C. Liu, *J. Mol. Struct.: THEOCHEM* **2002**, *588*, 1.

[12]	J. Tomasi, B. Mennucci, R. Cammi, *Chem. Rev.* **2005**, *105*, 2999.

[13]	T. Lu, F. Chen, *J. Comput. Chem.* **2012**, *33*, 580.

[14]	W. Humphrey, A. Dalke, K. Schulten, *J. Mol. Graphics* **1996**, *14*, 33.

[15]	K. Momma, F. Izumi, *J. Appl. Crystallogr.* **2011**, *44*, 1272.

[16]	Z. Chen, C. S. Wannere, C. Corminboeuf, R. Puchta, P. V. R. Schleyer, *Chem. Rev.* **2005**, *105*, 3842.

[17]	H. Fallah-Bagher-Shaidaei, C. S. Wannere, C. Corminboeuf, R. Puchta, P. V. R. Schleyer, *Org. Lett.* **2006**, *8*, 863.

[18]	D. Geuenich, K. Hess, F. Köhler, R. Herges, *Chem. Rev.* **2005**, *105*, 3758.

[19]	Y. Guo, W. Wang, K. Guo, X. Chen, M. Wang, Z. Huang, Y. Zhu, W. Song, S. Jiao, *Nat Commun* **2025**, *16*, 2794.

[20]	Z.-Q. Lin, G. Fan, T. Zhang, F. Huo, Q. Xi, J. Wang, T. She, X. Zeng, J. Weng, W. Huang, *Adv. Energy Mater.* **2023**, *13*, 2203532.

[21]	H. Peng, S. Huang, V. Montes-García, D. Pakulski, H. Guo, F. Richard, X. Zhuang, P. Samorì, A. Ciesielski, *Angew. Chem. Int. Ed.* **2023**, *135*, e202216136.

[22]	M. Liang, N. Liu, X. Zhang, Y. Xiao, J. Yang, F. Yu, J. Ma, *Adv. Funct. Mater.* **2022**, *32*, 2209741.

[23]	H. Cui, J. Zhu, R. Zhang, S. Yang, C. Li, Y. Wang, Y. Hou, Q. Li, G. Liang, C. Zhi, *J. Am. Chem. Soc.* **2024**, *146*, 15393.

[24]	C. Su, H. He, L. Xu, K. Zhao, C. Zheng, C. Zhang, *J. Mater. Chem. A* **2017**, *5*, 2701.

[25]	M. Fu, Y. Chen, W. Jin, H. Dai, G. Zhang, K. Fan, Y. Gao, L. Guan, J. Chen, C. Zhang, J. Ma, C. Wang, *Angew. Chem. Int. Ed.* **2024**, *63*, e202317393.

[26]	Y. Guo, W. Wang, K. Guo, X. Chen, M. Wang, Z. Huang, Y. Zhu, W. Song, S. Jiao, *Nat. Commun.* **2025**, *16*, 2794.

[27]	T. Lu, Q. Chen, *Chem. Methods* **2021**, *1*, 231.

[28]	Y. Li, P. Ren, Y. Zhang, S. Wang, J. Zhang, P. Yang, A. Liu, G. Wang, Z. Chen, M. An, *J. Ind. Eng. Chem.* **2023**, *118*, 78.

[29]	M. Alonso, B. Pinter, T. Woller, P. Geerlings, F. De Proft, *Comput. Theor. Chem.* **2015**, *1053*, 150.

[30]	Y. Gholiee, *Supramol. Chem.* **2024**, *35*, 7.

[31]	N. Wang, Z. Guo, Z. Ni, J. Xu, X. Qiu, J. Ma, P. Wei, Y. Wang, *Angew Chem Int Ed* **2021**, *60*, 20826.

[32]	Y. Lu, C.-Z. Zhao, J.-Q. Huang, Q. Zhang, *Joule* **2022**, *6*, 1172.

[33]	J. Chen, E. Quattrocchi, F. Ciucci, Y. Chen, *Chem* **2023**, *9*, 2267.

[34]	J. Xie, F. Yu, J. Zhao, W. Guo, H.-L. Zhang, G. Cui, Q. Zhang, *Energy Storage Mater.* **2020**, *33*, 283.

[35]	W. Wang, R. Li, Z. Duan, J. Zhao, Y. Qi, Q. Guo, Q. Peng, D. Wang, S. Han, L. Zhang, *Chem. Eng. J.* **2023**, *456*, 141019.

[36]	L. Zhang, I. A. Rodríguez‐Pérez, H. Jiang, C. Zhang, D. P. Leonard, Q. Guo, W. Wang, S. Han, L. Wang, X. Ji, *Adv. Funct. Mater.* **2019**, *29*, 1902653.

[37]	Q. Wu, X. Tang, Y. Qian, J. Duan, R. Wang, J. Teng, J. Li, *ACS Appl. Energy Mater.* **2021**, *4*, 10234.

[38]	W. Chen, K. Wang, X. He, X. Chen, T. Huang, J. Chen, W. Huang, X. Yang, X. Ren, X. Ouyang, J. Liu, F. Pan, B. Xiao, Q. Zhang, J. Hu, *Nano Energy* **2025**, *139*, 110950.

[39]	H. Zhang, L. Zhong, J. Xie, F. Yang, X. Liu, X. Lu, *Adv. Mater.* **2021**, *33*, 2101857.

[40]	C. Zhang, W. Ma, C. Han, L.-W. Luo, A. Daniyar, S. Xiang, X. Wu, X. Ji, J.-X. Jiang, *Energy Environ. Sci.* **2021**, *14*, 462.

[41]	C. Zhijiang, H. Chengwei, *J. Power Sources* **2011**, *196*, 10731.

[42]	K. Koshika, N. Sano, K. Oyaizu, H. Nishide, *Macromol. Chem. Phys.* **2009**, *210*, 1989.

[43]	A. Sethi, A. Kumar U., V. M. Dhavale, *ChemPhysChem* **2023**, *24*, e202300098.

[44]	X. Wu, Y. Xu, C. Zhang, D. P. Leonard, A. Markir, J. Lu, X. Ji, *J. Am. Chem. Soc.* **2019**, *141*, 6338.

[45]	S. Li, Z. Zhang, J. Wu, X. Guo, Y. Chen, C. Wang, F. Yu, Z. Wang, D. Li, Y. Chen, *J. Power Sources* **2023**, *560*, 232691.

[46]	H. Jiang, X. Ji, *Carbon Energy* **2020**, *2*, 437.

[47]	Q. Guo, K.-I. Kim, S. Li, A. M. Scida, P. Yu, S. K. Sandstrom, L. Zhang, S. Sun, H. Jiang, Q. Ni, D. Yu, M. M. Lerner, H. Xia, X. Ji, *ACS Energy Lett.* **2021**, *6*, 459.

[48]	Q. Guo, K. Kim, H. Jiang, L. Zhang, C. Zhang, D. Yu, Q. Ni, X. Chang, T. Chen, H. Xia, X. Ji, *Adv. Funct. Mater.* **2020**, *30*, 2002825.

[49]	Z. Song, W. Liu, Q. Huang, Y. Lv, L. Gan, M. Liu, *Chem. Sci.* **2025**, *16*, 16542.

[50]	H. Zhang, H. Wang, Y. Huang, J. Yan, Z. Meng, J. Xie, C. Li, Y. Ma, Y. Min, *Chem. Eng. J.* **2026**, *527*, 172149.

[51]	T. Shi, Z. Song, C. Hu, Q. Huang, Y. Lv, L. Miao, L. Gan, D. Zhu, M. Liu, *Angew. Chem. Int. Ed.* **2025**, *64*, e202501278.

[52]	Y. Zhang, M. Li, Z. Li, Y. Lu, H. Li, J. Liang, X. Hu, L. Zhang, K. Ding, Q. Xu, H. Liu, Y. Wang, *Angew Chem Int Ed* **2024**, *63*, e202410342.

[53]	U. Mittal, F. Colasuonno, A. Rawal, M. Lessio, D. Kundu, *Energy Storage Materials* **2022**, *46*, 129.

[54]	H. Glatz, E. Lizundia, F. Pacifico, D. Kundu, *ACS Appl. Energy Mater.* **2019**, *2*, 1288.

[55]	Y. Luo, F. Zheng, L. Liu, K. Lei, X. Hou, G. Xu, H. Meng, J. Shi, F. Li, *ChemSusChem* **2020**, *13*, 2239.

[56]	Z. Chen, H. Cui, Y. Hou, X. Wang, X. Jin, A. Chen, Q. Yang, D. Wang, Z. Huang, C. Zhi, *Chem* **2022**, *8*, 2204.

[57]	X. Liu, J. Shang, Y. Cheng, Q. Pan, J. Li, C.-H. Liu, H. Liu, F. Zhang, Y.-Y. Zhu, T. Liu, Y. Tang, *Angew. Chem. Int. Ed.* **2025**, *64*, e202511229.

[58]	W. Li, X. Li, Y. Gu, B. Zhu, Y. Zheng, J. Shi, W. Tang, *Mater. Today* **2025**, *88*, 229.

[59]	T. Wu, Y. Liu, J. Ye, Y. Chen, G. Dai, X. Zhang, Y. Zhao, *J. Power Sources* **2024**, *612*, 234779.

[60]	X. Wang, G. Li, Y. Han, F. Wang, J. Chu, T. Cai, B. Wang, Z. Song, *Chemsuschem* **2021**, *14*, 3174.

[61]	W. Ma, L.-W. Luo, P. Dong, P. Zheng, X. Huang, C. Zhang, J.-X. Jiang, Y. Cao, *Adv. Funct. Mater.* **2021**, *31*, 2105027.

[62]	F. Otteny, V. Perner, D. Wassy, M. Kolek, P. Bieker, M. Winter, B. Esser, *ACS Sustainable Chem. Eng.* **2020**, *8*, 238.

[63]	C. N. Gannett, B. M. Peterson, L. Shen, J. Seok, B. P. Fors, H. D. Abruña, *Chemsuschem* **2020**, *13*, 2428.

[64]	M. Kolek, F. Otteny, P. Schmidt, C. Mück-Lichtenfeld, C. Einholz, J. Becking, E. Schleicher, M. Winter, P. Bieker, B. Esser, *Energy Environ. Sci.* **2017**, *10*, 2334.

[65]	J. D. Henderson, S. D. C. Buchanan, L. H. Grey, M. C. Biesinger, *Appl. Surf. Sci.* **2026**, *730*, 166284.

[66]	J. Winiarski, W. Tylus, K. Winiarska, I. Szczygieł, B. Szczygieł, *J. Spectro.* **2018**, *2018*, 1.

[67]	J. H. Fox, J. D. Nuttall, T. E. Gallon, *Surface Science* **1977**, *63*, 390.

[68]	P. Blumentrit, M. Yoshitake, S. Nemšák, T. Kim, T. Nagata, *Appl. Surf. Sci.* **2011**, *258*, 780.

[69]	F. Cocco, B. Elsener, M. Fantauzzi, D. Atzei, A. Rossi, *RSC Adv.* **2016**, *6*, 31277.

[70]	J. P. Merrick, D. Moran, L. Radom, *J. Phys. Chem. A* **2007**, *111*, 11683.

[71]	Z. Tang, H. Zhu, Z. Pan, J. Gao, J. Zhang, *Phys. Chem. Chem. Phys.* **2024**, *26*, 17549.

[72]	R. Pal, S. G. Patra, P. K. Chattaraj, *J. Chem. Sci.* **2022**, *134*, 108.

[73]	M. Cui, N. Ma, H. Lei, Y. Liu, W. Ling, S. Chen, J. Wang, H. Li, Z. Li, J. Fan, Y. Huang, *Angew. Chem. Int. Ed.* **2023**, *62*, e202303845.

[74]	L. Cheng, C. Liu, D. Han, S. Ma, W. Guo, H. Cai, X. Wang, *J. Alloys Compd.* **2019**, *774*, 255.

[75]	J. Winiarski, W. Tylus, B. Szczygieł, *Appl. Surf. Sci.* **2016**, *364*, 455.

[76]	M. Mouanga, P. Berçot, J. Y. Rauch, *Corros. Sci.* **2010**, *52*, 3984.
